# Control of Hox transcription factor concentration and cell-to-cell variability by an auto-regulatory switch

**DOI:** 10.1242/dev.168179

**Published:** 2019-01-25

**Authors:** Dimitrios K. Papadopoulos, Kassiani Skouloudaki, Ylva Engström, Lars Terenius, Rudolf Rigler, Christoph Zechner, Vladana Vukojević, Pavel Tomancak

**Affiliations:** 1 Max-Planck Institute of Molecular Cell Biology and Genetics, 01307 Dresden, Germany; 2 Department of Molecular Biosciences, The Wenner-Gren Institute, Stockholm University, 10691 Stockholm, Sweden; 3 Center for Molecular Medicine (CMM), Department of Clinical Neuroscience, Karolinska Institutet, 17176 Stockholm, Sweden; 4 Department of Medical Biochemistry and Biophysics, Karolinska Institutet, 17177 Stockholm, Sweden; 5 Laboratory of Biomedical Optics, Swiss Federal Institute of Technology, 1015 Lausanne, Switzerland; 6 Center for Systems Biology Dresden, 01307 Dresden, Germany

**Keywords:** Auto-regulation, Fluorescence correlation spectroscopy, Hox genes, Protein noise, Transcription factors, Variability

## Abstract

The variability in transcription factor concentration among cells is an important developmental determinant, yet how variability is controlled remains poorly understood. Studies of variability have focused predominantly on monitoring mRNA production noise. Little information exists about transcription factor protein variability, as this requires the use of quantitative methods with single-molecule sensitivity. Using Fluorescence Correlation Spectroscopy (FCS), we have characterized the concentration and variability of 14 endogenously tagged TFs in live *Drosophila* imaginal discs. For the Hox TF Antennapedia, we investigated whether protein variability results from random stochastic events or is developmentally regulated. We found that Antennapedia transitioned from low concentration/high variability early, to high concentration/low variability later, in development. FCS and temporally resolved genetic studies uncovered that Antennapedia itself is necessary and sufficient to drive a developmental regulatory switch from auto-activation to auto-repression, thereby reducing variability. This switch is controlled by progressive changes in relative concentrations of preferentially activating and repressing Antennapedia isoforms, which bind chromatin with different affinities. Mathematical modeling demonstrated that the experimentally supported auto-regulatory circuit can explain the increase of Antennapedia concentration and suppression of variability over time.

## Abstract (precis)

**Summary:** Preferentially repressing and activating isoforms of the Hox transcription factor Antennapedia elicit a developmental regulatory switch from auto-activation to auto-repression that increases concentration and suppresses cell-to-cell variability over time.

## INTRODUCTION

In order to understand the mechanisms that control pattern formation and cell fate specification in developing organisms, the intranuclear concentration, DNA-binding kinetics and cell-to-cell variability of relevant transcription factors (TFs) need to be quantified. TF concentration variability at the tissue level is thought to arise from diverse processes, including mRNA transcription, translation and protein degradation. Intrinsic noise is due to stochastic binding and interactions of proteins involved in transcriptional activation of a specific gene (Blake et al., 2003; Elowitz et al., 2002). Extrinsic noise arises from inter-cellular differences in abundance of the transcriptional and post-transcriptional machinery (Swain et al., 2002).

In undifferentiated tissue or cells, TF cell-to-cell variability can be the driving force for differentiation. For example, progressive establishment of a Nanog salt-and-pepper expression pattern leads to the formation of primitive endoderm in the mouse preimplantation embryo, whereas loss of the variability results in embryos lacking primitive endoderm entirely (Kang et al., 2013).

Conversely, in already differentiated tissue or cells, TF expression variability among cells may need to be counteracted to ensure homogeneity of gene expression patterns and robustness of commitment to a certain transcriptional regime. Examples are the Snail (Sna) TF, which is required for the invagination of the mesoderm during *Drosophila* gastrulation (Boettiger and Levine, 2013), or the Bicoid (Bcd) and Hunchback (Hb) TFs during early embryogenesis (Gregor et al., 2007a,b; Little et al., 2013).

In addition, differential cell fates within the same developmental territory may be specified by TFs deploying different DNA-binding dynamics, despite the existence of very similar concentrations (i.e. low variability). For example, studies on the Oct4 TF in early mouse embryos have shown that differential kinetic behavior of DNA binding, despite equal Oct4 concentration among blastomeres, ultimately dictates an early developmental bias towards lineage segregation (Kaur et al., 2013; Plachta et al., 2011).

So far, studies of gene expression variability have focused predominantly on monitoring the noise of mRNA production (Holloway et al., 2011; Holloway and Spirov, 2015; Little et al., 2013; Lucas et al., 2013; Paré et al., 2009). Little information exists about TF variability at the protein level within a tissue. Such studies require the use of quantitative methods with single-molecule sensitivity.

We have previously used Fluorescence Correlation Spectroscopy (FCS) to quantitatively characterize Hox TF interactions with chromatin in living salivary gland cells (Papadopoulos et al., 2015; Vukojevic et al., 2010). FCS is instrumental for quantifying TF dynamics in living cells or tissue (Clark et al., 2016; Kaur et al., 2013; Lam et al., 2012; Mistri et al., 2015; Papadopoulos et al., 2015; Perez-Camps et al., 2016; Szaloki et al., 2015; Tiwari et al., 2013; Tsutsumi et al., 2016). However, in these studies, only mobility has been measured for overexpressed proteins. To understand TF behavior *in vivo*, proteins need to be quantified at endogenous levels (Lo et al., 2015).

In this study, we take advantage of the availability of fly toolkits, in which TFs have been endogenously tagged using different methodologies, fosmid (Baumgartner et al., 1996), BAC (deposition of lines of Rebecca Spokony and Kevin White to FlyBase and the Bloomington Stock Center), FlyTrap (Buszczak et al., 2007; Kelso et al., 2004; Morin et al., 2001; Quinones-Coello et al., 2007) and MiMIC lines (Nagarkar-Jaiswal et al., 2015; Venken et al., 2011), to measure the intranuclear concentration of various TFs *in vivo* by FCS, and their cell-to-cell variability in fly imaginal discs. Imaginal discs are flat, single-layered epithelia comprising small diploid cells and many TFs are expressed in defined regions within these tissues during development.

## RESULTS

### Characterization of average protein concentrations and cell-to-cell variability of *Drosophila* TFs

**Fig. 1.**
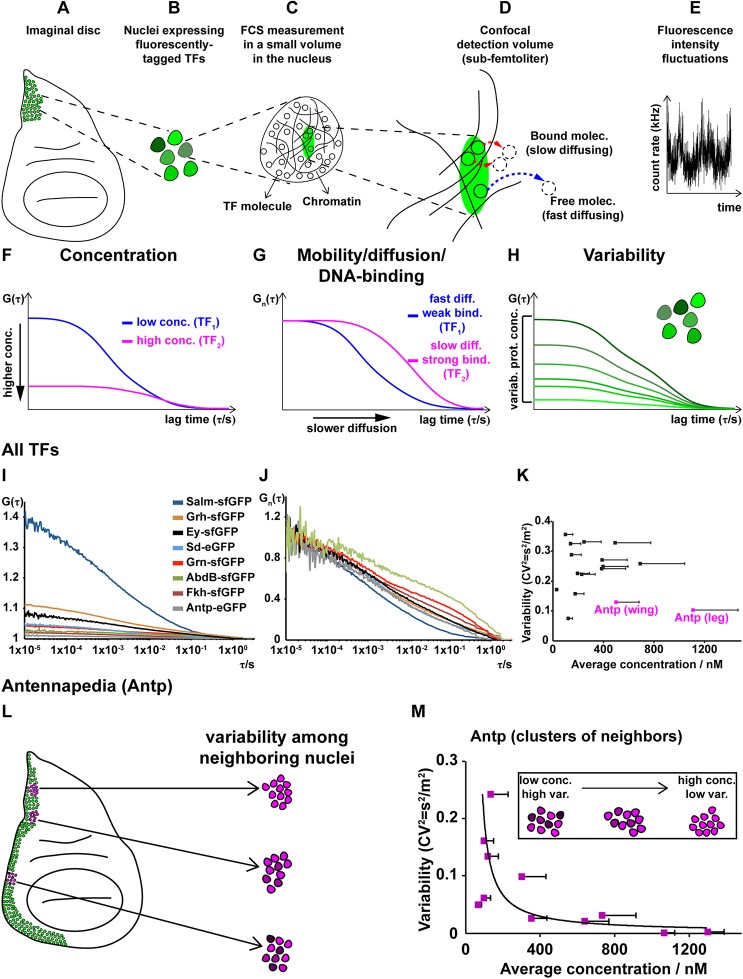
**Concentration, DNA-binding dynamics and cell-to-cell protein concentration variability of 14 *Drosophila* TFs.** (A-H) Workflow of the study of TFs by FCS (see Materials and Methods and supplementary Materials and Methods). (A) Schematic of an imaginal disc with cells expressing an endogenously-tagged TF (green), as imaged by confocal laser scanning microscopy. (B) Schematic of cell nuclei in neighboring cells expressing the TF at different concentrations. (C) Schematic of a cell nucleus with the observation volume element (OVE) for FCS measurements in the form of a prolate ellipsoid depicted as a green ellipse. (D) Magnified drawing of the OVE shown in C and its immediate surrounding. (E) Fluorescence intensity fluctuations occurring at fast and slow timescales are generated by TF molecules quickly/slowly diffusing into/out of the OVE. (F) After deploying temporal autocorrelation analysis to examine the fluorescence intensity fluctuations, temporal autocorrelation curves (henceforth referred to as FCS curves) are generated, which, after fitting with an appropriate model function, yield information about the absolute concentration and diffusion of TFs, as well as the fraction of fast- and slow-diffusing TF molecules. The concentration of molecules is inversely proportional to the *y*-axis amplitude of the FCS curve at zero lag time. (G) FCS curves normalized to the same amplitude. Processes that slow down the diffusion of TF molecules, such as binding to very large molecules (e.g. chromatin), are visible by a shift of the FCS curves to longer lag times. (H) FCS curves recorded in neighboring cell nuclei allow the calculation of protein concentration variability at the live tissue level. Here, the TF concentration is the highest in the brightest green nucleus, corresponding to the FCS curve with the lowest amplitude, and the TF concentration is the lowest in the dark green nucleus, corresponding to the FCS curve with the highest amplitude. (I) Representative average FCS measurements of eight TFs. (J) FCS curves shown in I, normalized to the same amplitude, *G*_*n*_(*τ*)=1 at *τ*=10 μs. (K) Variability of the 14 TFs as a function of concentration. (L) Variability in concentration of endogenous Antp in the wing disc. Antp protein distribution in third instar wing imaginal disc (green). Examples of different regions in the disc, analyzed by FCS (magenta), displaying different variabilities in the concentration of nuclear Antp (darker and lighter magenta shades). (M) Variability of Antp concentration in clusters of neighboring cell nuclei as a function of its average concentrations. Error bars in K and M represent 1 s.d.

**Fig. 2.**
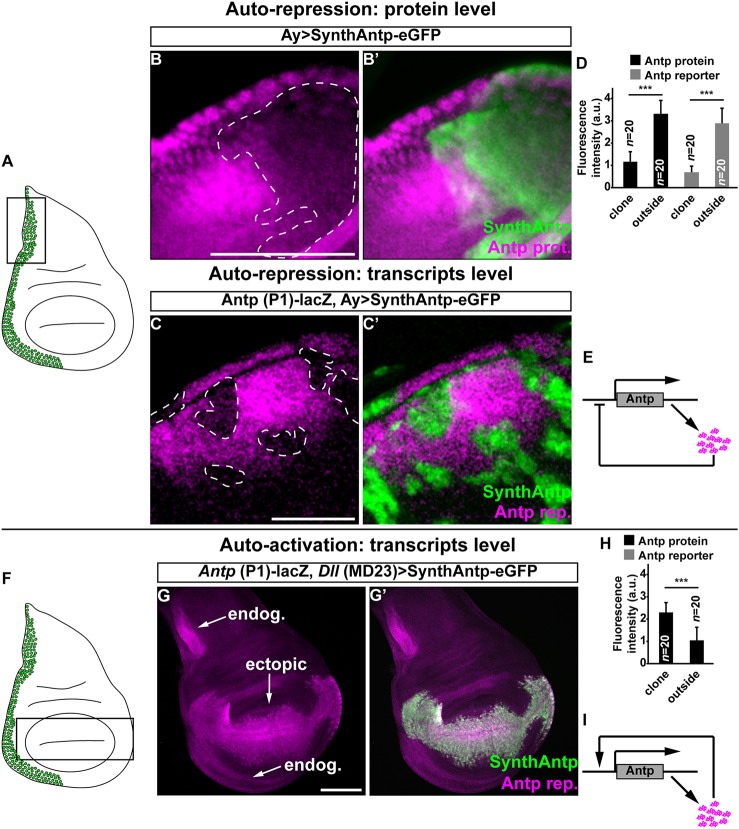
***Antp* activates and represses its own transcription.** (A) Schematic representation of the wing disc region of highest Antp expression (green cells). Antp is highly expressed in the wing disc in the regions of the notum that correspond to the structure of the prescutum in the adult cuticle, as well as in the base of the wing blade, which gives rise to the mesopleura and pteropleura of the adult thoracic cuticle. The black rectangle indicates the region of clonal analysis in B-C′. (B,B′) Clonal overexpression of a *SynthAntp-eGFP* construct. Dashed line in B shows a clone in the Antp expression domain. (C,C′) Transcriptional auto-repression of Antp using the *Antp* P1-*lacZ*. (D) Quantification of repression of Antp protein and reporter inside the repression clones, when compared with the surrounding tissue. (E) Schematic of Antp transcriptional auto-repression. Repression can be direct or indirect. (F) Wing disc region of ectopic Antp P1 reporter expression in G,G′. (G,G′) Ectopic induction of *Antp* P1-*lacZ* in distal compartments of the wing disc by expression of *SynthAntp-eGFP* using *Dll*-Gal4 (MD23). (H) Quantification of auto-activation of Antp reporter within the *Dll*-Gal4 expression domain when compared with the surrounding tissue. (I) Schematic representation of Antp auto-activation. Scale bars: 100 μm.

Average concentrations of TFs in neighboring nuclei of third instar imaginal discs were measured by FCS (Fig. 1A-J and Fig. S1A-P). FCS is a non-invasive method with single-molecule sensitivity, in which a confocal arrangement of optical elements is used to generate a small (sub-femtoliter) detection volume inside living cells, from which fluorescence is being detected (Fig. 1C,D; green ellipsoid). Fluorescent molecules diffuse through this observation volume, yielding fluorescence intensity fluctuations that are recorded over time by detectors with single-photon sensitivity (Fig. 1E). These fluctuations are subsequently subjected to temporal autocorrelation analysis, yielding temporal autocorrelation curves (henceforth referred to as FCS curves, Fig. 1F), which are then fitted with selected models to extract quantitative information about the dynamic processes underlying the generation of the recorded fluctuations. In the case of molecular movement of TFs (see supplementary Materials and Methods), information can be obtained regarding: (1) the absolute TF concentrations (Fig. 1F); (2) TF dynamic properties, such as diffusion times, differences in their interactions with chromatin and fractions of free-diffusing versus chromatin-bound TFs (Fig. 1G); and (3) cell-to-cell TF concentration variability (Fig. 1H).

We selected 14 TFs based on the availability of homozygous endogenously tagged transgenes and on the generation of robust fluorescence in distinct patterns in various imaginal discs. For the 14 TFs, we measured average concentrations ranging across about two orders of magnitude among the different TFs, from ∼30 nM to ∼1.1 μM (∼400 to 15,500 molecules per nucleus, respectively) (Fig. 1I, Fig. S1A-Q and supplementary Materials and Methods). Various diffusion times and fractions of slow- and fast-diffusing TF molecules (Fig. 1J) indicated differential mobility and degree of DNA-binding among different TFs (Vukojevic et al., 2010). Comparison of the *y*-axis amplitudes at the zero lag time of the FCS curves, which are inversely proportional to the concentration of fluorescent molecules (Fig. 1F), provides information about concentration variability (heterogeneity) among different cell nuclei, i.e. reflects the heterogeneity of protein concentration at the tissue level (Fig. 1H). For all 14 TFs studied, the variability, expressed as the variance over the mean squared, 
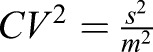
, was determined to be in the range 7−37% (Fig. 1K and Fig. S1Q).

In biological systems, the Fano factor, which is expressed as the variance over the mean (
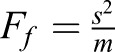
, in concentration units), is a commonly used index to quantify variability. It has been proposed that Fano factor values that increase with average concentrations indicate that the underlying transcriptional processes cannot be sufficiently explained by a simple one-step promoter configuration with purely intrinsic Poissonian noise and that extrinsic noise is likely to contribute significantly to the overall variability (Newman et al., 2006; Schwanhäusser et al., 2011; Taniguchi et al., 2010). For all TFs measured, Fano factor values from 0 to 20 were obtained (Fig. S1R), in line with Fano factor values of other TFs determined previously to lie between 0 and 30 (Sanchez et al., 2011). Moreover, the majority of TFs examined show Fano factor values, F_f_>1, suggesting that transcriptional bursting is likely to be a significant source of the observed cell-to-cell variability. We used this dataset as a starting point for studying the control of variability during imaginal disc development.

The average concentration and variability of the investigated TFs showed no obvious interdependence (Fig. 1K), suggesting that if variability is controlled, there is not one control mechanism that is common to all investigated TFs. Among the studied TFs, the Hox protein Antennapedia (Antp) showed low variability (*CV*^2^<0.2) in high average concentrations, in particular in the leg disc (Fig. 1K). As low variability at the tissue level is likely to be achieved through regulatory mechanisms, we investigated Antp variability further by FCS. Because FCS performs best at low to moderate expression levels (see supplementary Materials and Methods), we performed this analysis in the wing disc where expression levels are lower than in the leg disc (Fig. 1K,L). We first established that the observed fluorescence intensity fluctuations were caused by diffusion of TF molecules through the confocal detection volume (Figs S2 and S1). FCS showed that different clusters of neighboring cells along the Antp expression domain in the wing disc display different average expression levels (Fig. 1L). Moreover, FCS showed that Antp cell-to-cell variability decreased with increasing Antp concentration (Fig. 1M), whereas the Fano factor increased (Fig. S1R). Such behavior is indicative of complex transcriptional regulatory processes (Franz et al., 2011; Smolander et al., 2011) that we further investigated using the powerful *Drosophila* genetic toolkit.

### Control of Antp concentration by transcriptional auto-regulation

One mechanism by which genes control their expression level variability is auto-regulation (Becskei and Serrano, 2000; Dublanche et al., 2006; Gronlund et al., 2013; Nevozhay et al., 2009; Shimoga et al., 2013; Thattai and van Oudenaarden, 2001). To test whether Antp can regulate its own protein levels, we monitored the concentration of endogenous Antp protein upon overexpression of *Antp* from a transgene. To distinguish between overexpressed and endogenous protein, we used synthetic Antp (SynthAntp) transgenes fused to eGFP (SynthAntp-eGFP). These transgenes encode the Antp protein (amino acids 278-378), which includes the homeodomain, the conserved YPWM motif and the C terminus (but lack the long and non-conserved N terminus of the protein, against which widely used Antp antibodies have been raised) and they harbor Antp-specific homeotic function (Papadopoulos et al., 2011). Clonal overexpression of *SynthAntp-eGFP* in the wing disc notum (Fig. 2A,B′,D and controls in Fig. S3D,D′) repressed the endogenous Antp protein, indicating that Antp is indeed able to regulate its own protein levels.

As Antp is a TF, we next asked whether the auto-repression occurs at the transcriptional level. The *Antp* locus is subject to complex transcriptional regulation, involving a distal and a proximal promoter (P1 and P2 promoters, respectively), spanning more than 100 kb of regulatory sequences. We established that the P1 promoter (rather than the P2 promoter) is predominantly required to drive expression of Antp in the wing disc notum (Fig. S3A-C′), in line with previous observations (Engstrom et al., 1992; Jorgensen and Garber, 1987; Zink et al., 1991) (see Materials and Methods). Moreover, mitotic recombination experiments in regions of the wing disc unique to P2 transcription have shown no function of the P2 promoter transcripts in wing disc development (Abbott and Kaufman, 1986). Thus, the P1 Antp reporter serves as a suitable reporter of the *Antp* locus transcriptional activity in this context.

Clonal overexpression of SynthAntp-eGFP in the wing disc repressed the *Antp* P1 transcriptional reporter (Fig. 2C,D and controls in Fig. S3E,E′). To rule out putative dominant-negative activity of the small SynthAntp-eGFP peptide, we also performed these experiments with the full-length Antp protein (Fig. S3F,F′) and found them to also repress the reporter. We conclude that the Antp protein is able to repress its own transcription from the P1 promoter (directly or indirectly), suggesting a possible mechanism of suppressing cell-to-cell variability of *Antp* expression levels (Fig. 2E).

In the course of these experiments, we noticed that ectopic overexpression of *SynthAntp-eGFP* or the full-length Antp protein from the *Distal-less* (*Dll*) (MD23) enhancer resulted in activation of the *Antp* P1 reporter in distal compartments of the wing disc, such as the wing pouch, where Antp is normally not detected (Fig. 2F-H and controls in Fig. S3G-H′). This suggests that as well as its auto-repressing function, Antp is also capable of activating its own transcription (Fig. 2I).

To exclude the possibility that the auto-activation and repression of Antp are artifacts of overexpression, we used FCS to measure the concentration of Antp triggered by different Gal4 drivers (Fig. S4A-E). We observed indistinguishable DNA-binding behavior by FCS, not only across the whole concentration range examined (Fig. S4F), but also between endogenous and overexpressed Antp (Fig. S5A,B). Importantly, the auto-activating and auto-repressing capacity of Antp was preserved even with the weak Gal4-driver *69B* (Fig. S4K,L) that triggered concentrations of Antp lower than its normal concentration in the leg disc (473 nM versus 1110 nM), indicating that auto-activation and auto-repression of *Antp* take place at endogenous protein concentrations. We conclude that *Antp* is able to repress and activate its own transcription (Fig. 2E,I), and hypothesize that this auto-regulatory circuit sets the ‘correct’ concentration of Antp protein in imaginal discs.

### A temporal switch controls the transition of *Antp* from a state of auto-activation to a state of auto-repression

**Fig. 3.**
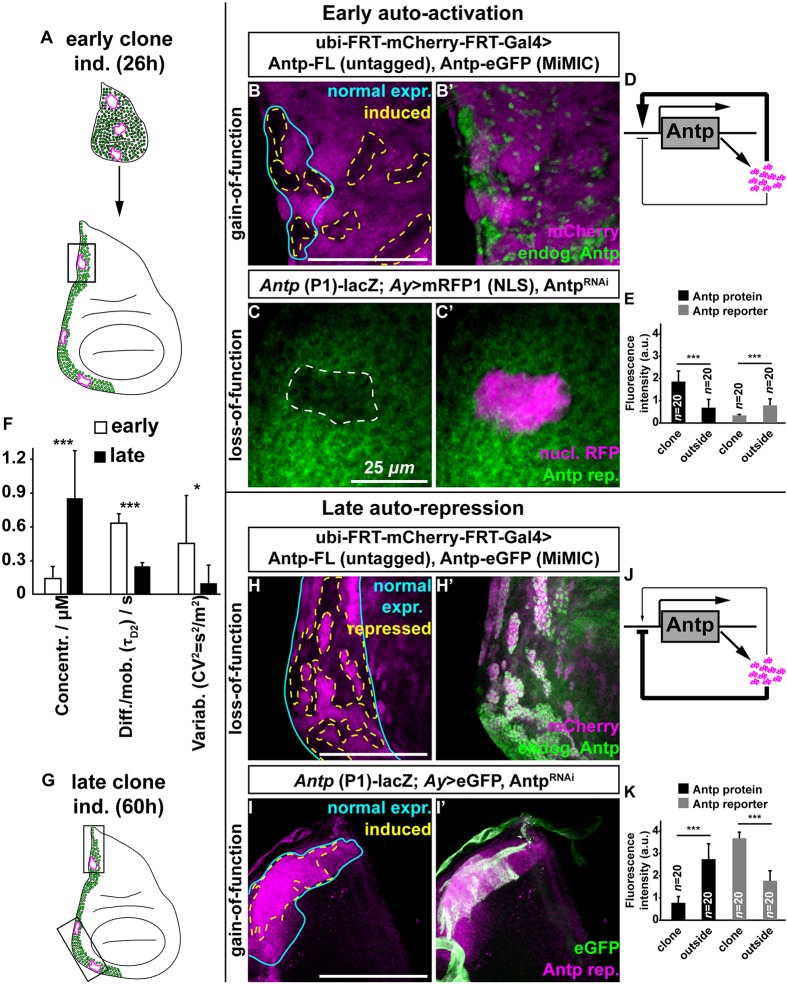
**Antp switches from transcriptional auto-activation to auto-repression.** (A) Clone induction at 26 h (early) with analysis at third instar larval stage (∼96-120 h of development). Black rectangle represents the corresponding region of clonal analysis. (B,B′) Early clonal induction of full-length, untagged *Antp*, (mCherry positive) reveals strong auto-activation of endogenous Antp-eGFP (dashed lines in B). The cyan line outlines the region of highest endogenous Antp expression. The whole *Antp* expression domain expresses *Antp-eGFP*, but overexpression clones (subregions marked by absence of mCherry staining) express *Antp-eGFP* much more strongly (B′). (C,C′) *Antp* P1 transcription in *Antp* RNAi knockdown clones (early clonal induction, dashed line in C) marked by nuclear mRFP1. (D) Updated *Antp* auto-activation model, showing strong auto-activation of Antp at early stages. (E) Quantification of fluorescence intensities (in a.u.) upon early clonal induction of endogenous Antp protein, upregulated upon overexpression of untagged full-length *Antp* (as in B,B′), and of *Antp* reporter downregulated upon knockdown by RNAi (as in C,C′). (F) Concentration, DNA-binding and variability studied by FCS at second instar leg and wing discs (FCS analysis in Fig. S8). Low concentration, low degree of DNA-binding and high variability are observed in second instar wing and leg discs, but the opposite is true for third instar discs. (G) Clone induction at 60 h (late) with analysis at third instar larval stage (∼96-120 h of development). Black rectangles represent the corresponding regions of clonal analysis. (H,H′) Late-induced clones (dashed lines in H), expressing full-length, untagged *Antp* (mCherry positive). Auto-repression of *Antp* in these regions is monitored by the endogenous Antp-eGFP protein. The cyan lines in H and I outline the region of strong endogenous expression of the Antp protein (H) and the *Antp* P1 reporter (I). (I,I′) *Antp* P1 transcription in late *Antp* RNAi knockdown clones (dashed line in I, 60 h of development) within the *Antp* normal expression domain, marked by nuclear mRFP1. Cytoplasmic eGFP marks the *Antp* knockdown clone (I′). (J) Updated *Antp* auto-repression model showing the pronounced auto-repressing capacity of Antp at late stages. (K) Quantification of fluorescence intensities (in a.u.) upon late clonal induction of endogenous Antp protein, downregulated upon overexpression of untagged full-length *Antp* (as in H,H′), and of *Antp* reporter upregulated upon knockdown by RNAi (as in I,I′). Scale bars: 100 μm.

To further investigate the mechanism by which the *Antp* auto-regulatory circuit sets the precise Antp expression levels, we next asked whether the seemingly opposing auto-regulatory activities of *Antp* are separated in time during development. To achieve this, we induced gain-of-function clones of full-length untagged *Antp* either at 26 h (first larval instar – henceforth referred to as ‘early’ stage) or at 60 h (late second larval instar – henceforth referred to as ‘late’ stage) of development and analyzed the clones in late third instar wing imaginal discs (Fig. 3). We chose these time points based on Antp expression being widespread during first instar disc development and therefore possibly amenable to auto-activation before becoming confined to the proximal disc regions, whereas in the late second instar it is restricted to proximal-only regions (Emerald and Cohen, 2004). As a pre-requisite for this analysis, we established that the *Antp-eGFP* homozygous viable MiMIC allele recapitulates the endogenous Antp pattern in the embryo and all thoracic imaginal discs, and therefore can be used to monitor endogenous Antp protein (Fig. S6). Clonal induction of full-length untagged *Antp* in early development triggered strong auto-activation of *Antp-eGFP* (Fig. 3A,B,B′ and quantification in E, see controls in Fig. S7A-C′). As before, we confirmed that early auto-activation of *Antp* is transcriptional and similar for both full-length and SynthAntp proteins (Fig. S7D-E′, see controls in F-G′). Early auto-activation was further supported by results from a loss-of-function experiment, where RNAi-mediated early knockdown of *Antp* resulted in downregulation of the *Antp* reporter (Fig. 3C,C′, see controls in Fig. S7H,H′). The loss- and gain-of-function analysis together suggest that during early disc development Antp is required for sustaining its own expression.

In contrast, clonal induction during the late second instar stage (Fig. 3G) repressed Antp-eGFP (Fig. 3H,H′ and quantification in K) and, reciprocally, the clonal knockdown by RNAi triggered auto-activation of Antp transcription (Fig. 3I,I′). Hence, in contrast to early development, Antp represses its own expression in third instar discs. Although the gain-of-function experiments show that Antp is sufficient to execute auto-regulation, loss-of-function analysis indicates that it is also necessary for both repression and activation at the transcriptional level.

Together, these results revealed the existence of a switch in Antp auto-regulatory capacity on its own transcription during development. Starting from a preferentially auto-activating state early in development (Fig. 3D), Antp changes to an auto-inhibitory mode at later developmental stages (Fig. 3J).

### During development, Antp switches from a low-concentration/high-variability to a high-concentration/low-variability state

If the *Antp* auto-repressive state limits the variability of Antp protein concentration among neighboring cells late in development, we expected that the variability would be higher during earlier stages, when auto-repression does not operate. We therefore used FCS to characterize the endogenous expression levels and cell-to-cell variability of Antp concentration in nuclei of second instar wing and leg discs. We observed significantly lower average concentrations of Antp protein in second versus third instar wing and leg discs, and the inverse was true for concentration variability (Fig. 3E and Fig. S8A,A′,C), indicating that the developmental increase in concentration is accompanied by suppression of concentration variability. In addition, FCS revealed a notable change in Antp characteristic decay times (signifying molecular diffusion, limited by chromatin binding) at early versus late stages (Fig. S8B). This behavior indicates that endogenous Antp is initially moving fast in the nucleus, as it undergoes considerably fewer interactions with chromatin compared with later stages where its interactions with chromatin are more frequent and longer lasting.

Taken together, our measurements show that *Antp* is expressed at relatively low and highly variable levels in early developing discs, when genetic evidence indicates auto-activation capacity on its own transcription. Later in development, when Antp has reached a state of higher average concentrations, auto-repression kicks in, resulting in considerably lower variability among neighboring cells.

### Dynamic control of Antp auto-regulation by different Antp isoforms

**Fig. 4.**
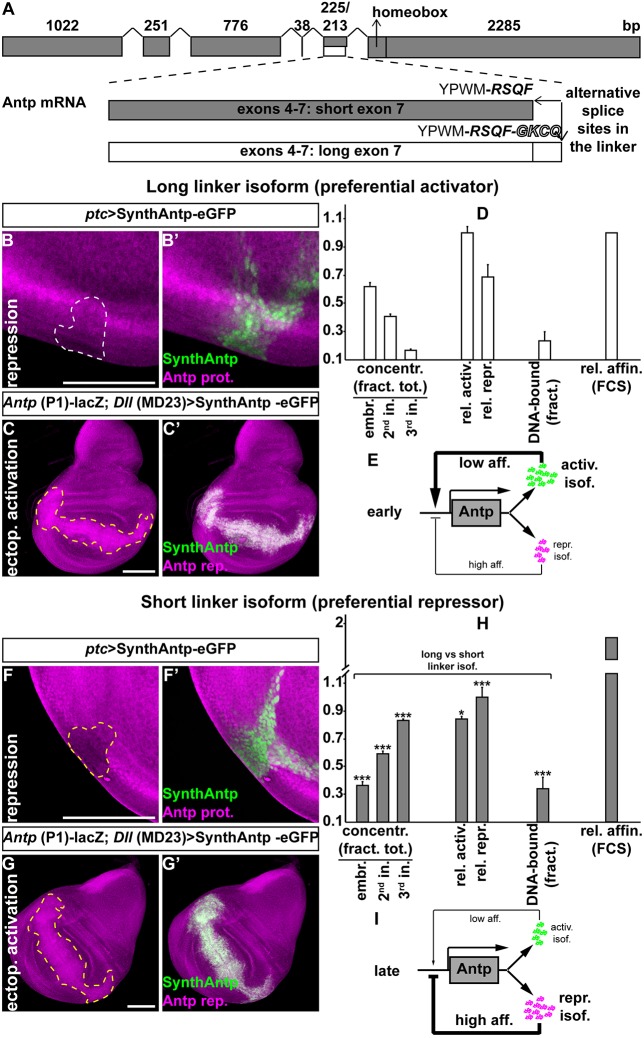
**Antp auto-activation and auto-repression relies on Antp isoforms with different binding affinities to chromatin.** (A) Schematic of the Antp mRNA, generated from the P1 promoter. Exons are represented by gray boxes. Magnified exons 4-7 (drawn to scale, omitting splicing points for simplicity) show the alternative splice site (3′ of exon 7), resulting in isoforms featuring a short linker between the YPWM motif and the homeodomain (RSQF, gray box) or a long linker isoform (RSQFGKCQ, white box). (B,B′) *SynthAntp-eGFP* bearing a long linker expressed by *ptc*-Gal4 and endogenous Antp protein auto-repression were monitored at the proximal region of the wing disc. A white dashed line outlines the region of auto-repression that was used for quantification (see Materials and Methods). (C,C′) Similar to B,B′, except that expression was induced by *Dll* (MD23)-Gal4 distally (yellow dashed line). (D) Abundance of long linker isoform (see Materials and Methods); auto-activation and auto-repression efficiencies (Materials and Methods); DNA-bound fractions, measured by FCS (Fig. S10); and relative affinity of binding to chromatin, calculated by FCS (Fig. S10) are presented for comparison with H. (E) Updated model of *Antp* auto-regulation. The activating isoform binds with lower affinity to the P1 Antp promoter, but is produced in excess, relative to the repressing isoform, resulting in preferential activation of transcription. (F-G′) Similar to B-C′ for the short linker isoform. (H) Similar to D for comparison. (I) Updated qualitative model representation of Antp repression as in E, whereby at later stages an excess of Antp auto-repressor accounts for negative feedback on transcriptional regulation of the P1 promoter, resulting in partial activation of transcription; hence, expression is maintained. Scale bars: 100 μm.

The changing binding behavior of Antp on chromatin from second to third instar discs and the developmental transition from an auto-activating to an auto-repressing state suggested a causal relationship between the two phenomena. We therefore sought to identify molecular mechanisms that could link the observed changes in Antp chromatin binding to *Antp* auto-activation and repression. It is well established that the *Antp* mRNA contains an alternative splice site in exon 7 immediately upstream of the homeobox-containing exon 8, and generates Antp isoforms differing in as little as four amino acids in the linker between the YPWM motif (a co-factor-interacting motif) and the homeodomain (Fig. 4A) (Stroeher et al., 1988). Our previous observation that long linker isoforms favor transcriptional activation of Antp target genes, whereas short linker isoforms favor repression of Antp targets (Papadopoulos et al., 2011), prompted us to examine whether the linker length is also responsible for differences in auto-regulation.

Ectopic expression of SynthAntp-eGFP peptides featuring a long linker displayed significantly weaker repression capacity on endogenous Antp, when compared with their short linker counterparts (Fig. 4B,B′,F,F′ and quantified in D,H, see also Materials and Methods). We confirmed that, also in this case, the repression was at the transcriptional level (Fig. S9I-J′). Inversely, long-linker Antp isoforms exhibited stronger activation of Antp reporter, when compared with short-linker isoforms (Fig. 4C,C′,G,G′ and quantified in D,H; see also Materials and Methods). We additionally validated that short-linker isoforms encoded by full-length or *SynthAntp* cDNAs behaved as weaker auto-activating and stronger auto-repressing Antp species in all our previous experiments using the endogenous Antp protein and the P1 reporter (Fig. S9A-H′). We conclude that, also in the case of *Antp* auto-regulation, short-linker isoforms function as more potent repressors, whereas long-linker isoforms operate as more potent activators.

As the *Antp* P1 promoter undergoes a switch from preferential auto-activation to auto-repression, and short- and long-linker Antp isoforms function as preferential auto-repressors and auto-activators, respectively, it appeared possible that the switch in *Antp* regulation is executed at the level of transcript variant abundance of these isoforms. Therefore, we next quantified the relative abundance of long- and short-linker transcript variants in the embryo, second and third instar discs (Fig. 4D,H). The data showed that the abundance of the long-linker variant decreased, whereas the abundance of the short-linker variant increased over time in development, in line with previous observations (Stroeher et al., 1988). Thus, as hypothesized, this finding suggests that relative transcript variant abundance may underlie the switch between auto-activation and auto-repression (without excluding additional mechanisms, such as changes in the chromatin modifications between early and later disc development, or the participation of different co-factors).

Relative changes in Antp transcript variant abundance (Fig. 4D,H), differential efficiency of their encoded isoforms to repress or activate the *Antp* gene (Fig. 4B-D,F-H), the developmental switch of *Antp* from auto-activation to repression (Fig. 3) and the different mobility of Antp between second and third instar imaginal discs (Fig. 3E) all pointed towards the hypothesis that the two isoforms have different modes of interaction with chromatin. To investigate this, we expressed the two isoforms from the *69B* enhancer in third instar wing and antennal discs. This results in Antp concentrations close to (if not below) endogenous levels (Fig. S4A-J). FCS measurements revealed that the short-linker isoform displayed longer characteristic decay times and a higher fraction of DNA-bound molecules, suggesting stronger and more pronounced binding to chromatin than its long-linker counterpart (Fig. 4D,H and Fig. S10A,B). With chromatin (and therefore Antp-binding sites configuration), as well as the presence of co-factor proteins, being identical between the two instances (short- and long-linker isoforms examined in third instar wing and antennal imaginal discs of the same age), we were able to directly compare the apparent equilibrium dissociation constants for the two isoforms (see supplementary Materials and Methods). We found that the affinity of binding to chromatin (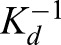) of the repressing short-linker isoform was at least 2.3 times higher compared with the activating long-linker isoform (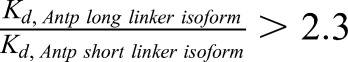) (Fig. 4D,H and Fig. S10C-D′). To corroborate these findings, we also performed gel-shift experiments to test how full-length recombinant Antp isoforms, which bear a short and a long linker, bind previously characterized Antp-binding sites. We found that equal amounts of Antp long-linker isoform bind *Antp*-binding sites more weakly than its short linker counterpart (Fig. S11). Collectively, these experiments support the notion that differences in Antp regulation during disc development can be largely attributed to differences in the affinity of the investigated Antp isoforms.

Taken together, the switch of Antp from an auto-activating to an auto-repressing state and the alteration of its DNA-binding behavior during disc development can be largely explained by a temporal developmental regulation of the relative concentrations of preferentially auto-activating and auto-repressing Antp protein isoforms. These isoforms display distinct properties in their modes of interaction with chromatin (Fig. 4E,I).

### Robustness of Antp auto-regulation

**Fig. 5.**
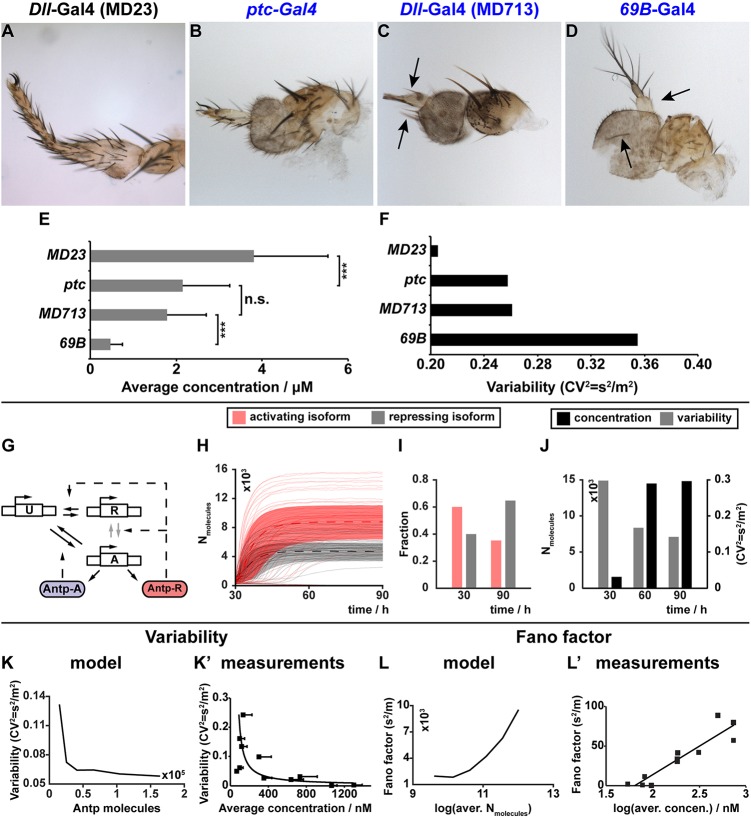
**Concentrations resulting in low variability are required for Antp homeotic function.** (A-D) Transformations of the distal antenna into a tarsus in adult flies, caused by *SynthAntp-eGFP* overexpression in antennal discs (Fig. S4A-D). Ectopic tarsi range from complete (A) to milder transformations of the arista (B,C) or ectopic leg bristles in the third antennal segment (C,D, arrows). (E,F) Measurements of SynthAntp concentration and cell-to-cell variability of antennal discs (Fig. S4A-D) in the corresponding antennal discs (A-D). The three Gal4 drivers (B-D) result in partial transformations, despite being expressed at similar levels to the wild-type Antp protein in the leg disc. However, their variability is higher than the endogenous variability (*CV*^2^=0.1). In contrast, Dll-Gal4 (MD23) results in much more robust homeotic transformations (A), accompanied by the lowest variability and closest to the wild-type condition. (G-J) A dynamic promoter, which drives transcription of *Antp* followed by a splicing step, leads to either the repressing (‘R’ in G) or activating (‘A’ in G) isoform of Antp. In the absence of Antp, the promoter is inactive and transcription cannot take place (‘U’ in G). This promoter configuration leads to suppression of variability and increase in Antp concentration (J). Trajectories of individual simulations are presented in H and the distribution of the Antp isoforms, predicted by the model, are presented in I. (K-L′) Model predictions (K,L) and experimental data validation (K′,L′) of variability (K) and protein Fano factor (L) as a function of Antp concentration.

The mechanism of developmental Antp auto-regulation offered a possible explanation for the observed increase in Antp concentration from second to third instar discs, as well as the suppression of variability. An unresolved issue is the functional significance of suppression of Antp variability in development. To test this, we need to manipulate variability, yet this is currently not possible to achieve at the endogenous locus. However, as average concentration and variability are interdependent, we used an ectopic expression system to progressively dampen Antp variability by manipulating its concentration. To achieve this, we expressed *SynthAntp* ectopically in the antennal disc, which is devoid of endogenous *Antp* expression, and monitored the extent (strength) of homeotic transformations induced by different Gal4 drivers corresponding to different SynthAntp concentrations (as measured by FCS previously in Fig. S4A-D). In this experiment, expression of SynthAntp is controlled by the Gal4 driver, independently of the *Antp* locus, therefore the phenotypic output does not depend on *Antp* auto-regulation. We observed that partial transformations of antennae to tarsi could be obtained with drivers expressing Antp at close to endogenous concentration [*ptc*-Gal4, *Dll*-Gal4 (MD713) and *69B*-Gal4 drivers, Fig. 5B-D and Fig. S4B-D]. Therefore, Antp can repress the antennal and launch the leg developmental program in the antennal disc at endogenous concentrations, although not robustly across the tissue (see supplementary Materials and Methods and Table S1 for analysis of the phenotypic classes). As expected, the three weak transformation phenotypes, elicited by *ptc*-, *Dll* (MD713)- and *69B*-Gal4 (Fig. 5B-D), were accompanied by high variability of SynthAntp concentration in developing discs (Fig. 5E,F). In contrast, strong expression of *SynthAntp* from the *Dll*-Gal4 (MD23) enhancer resulted in robust homeotic transformation to a complete tarsus (Fig. 5A), accompanied by low cell-to-cell variability (Fig. 5F). This condition resembled most closely the endogenous Antp variability in the leg disc (*CV*^2^=0.103). Importantly, endogenous Antp and Antp overexpressed by any of the Gal4 drivers showed indistinguishable chromatin-binding behavior by FCS (Figs S4F and S5A,B). Therefore, robust *Antp* homeotic function can be achieved at concentrations that are accompanied by low variability.

In order to further substantiate the qualitative model of Antp auto-regulation suggested by our findings and examine its impact on protein variability, we developed a simple mathematical model of stochastic *Antp* expression (see supplementary Materials and Methods and Table S2). This model tests whether positive and negative auto-regulation of *Antp* through distinct isoforms is sufficient to explain the increase in protein concentration and decrease in nucleus-to-nucleus variability from early to late stages. The model consists of a dynamic promoter, which drives transcription of *Antp* followed by a splicing step, yielding either the auto-repressing or the auto-activating isoform of Antp. As the repressing isoform has higher abundance at later stages, we assumed that splicing is more likely to generate this isoform than the activating isoform. The initial imbalance of Antp towards the activating isoform (Fig. 4D,H) is modeled through appropriate initial concentrations of each isoform.

As Antp copy numbers per nucleus are in the thousands at both early and late stages of development, the intrinsic noise of gene expression is likely to explain only a specific part of the overall variability in Antp concentrations (Elowitz et al., 2002; Taniguchi et al., 2010). The remaining extrinsic variability is due to cell-to-cell differences in certain factors affecting gene expression such as the ribosomal or ATP abundances. To check whether extrinsic variability significantly affects *Antp* expression, we expressed nuclear *mRFP1* constitutively, alongside endogenous *Antp-eGFP*, and measured their abundances (Fig. S12). With extrinsic factors affecting both genes similarly, we expected a correlation between the concentration of nuclear mRFP1 and Antp-eGFP. Our data showed a statistically significant correlation between mRFP1 and Antp (Fig. S11C, *r*=0.524 and *P*=9.77 10^−5^). Correspondingly, we accounted for extrinsic variability also in our model by allowing gene expression rates to randomly vary between cells (Zechner et al., 2012).

The promoter itself is modeled as a Markov chain with three distinct transcriptional states. In the absence of Antp, the promoter is inactive and transcription cannot take place (state ‘U’ in Fig. 5G). It can switch into a highly expressing state ‘A’ at a rate that is assumed to be proportional to the concentration of the auto-activating isoform (Antp-A, Fig. 5G). This resembles the positive auto-regulatory function of Antp. Conversely, the promoter can be repressed by recruitment of the auto-repressing isoform, state ‘R’ in the model (Antp-R, Fig. 5G). As the auto-repressing isoform of Antp can also activate the promoter, albeit significantly weaker than the auto-activating isoform, and vice versa, we allow the promoter to switch between states ‘A’ and ‘R’.

In this promoter model, it remains unclear whether the two isoforms compete for the same binding sites on the P1 promoter. In this case, an increase in concentration of repressing Antp species enhances the probability to reach state ‘R’ only if the promoter is in state ‘U’ (Fig. 5G). In the absence of competitive binding, the rate of switching between ‘A’ and ‘R’ also depends on the concentration of repressing isoforms of Antp (Fig. 5G, compare with Fig. S13A). We analyzed both model variants by forward simulation and found that both of them can explain the switch-like increase in average Antp concentration between early and late stages (Fig. 5J, compare with Fig. S13D) and the relative fraction of repressing and activating isoforms (Fig. 5I, compare with Fig. S13C). However, only the non-competitive binding model (Fig. 5G) can explain the substantial reduction of total Antp variability between early and late stages (Fig. 5J, Fig. S13D). Simulation trajectories of individual nuclei indicated an initial increase and a subsequent stabilization of concentration, whereas in the competitive model, or in the absence of the negative feedback, this is not achieved (Fig. 5H, compare with Fig. S13B,F). Additionally, we established that the negative feedback is required for suppression of variability (Fig. S13E,H), as otherwise no suppression of variability is conferred (Fig. S13H). Thus, the model suggested that auto-repression is required and that isoforms do not compete for binding to the P1 promoter.

To further validate this model, we analyzed how Antp variability scales with average concentrations, compared with our experimental measurements. To generate different average concentrations, we varied the gene expression rates over three orders of magnitude. The model predicted a decrease in variability as a function of total Antp concentration and an increase in the Fano factor. These findings are in good agreement with the experimental data (compare Fig. 5K with K′ and L with L′).

We next analyzed the model behavior under different genetic perturbations. Increase of Antp concentration by overexpressing *SynthAntp* transgenes (bearing either a long or a short linker isoform) from the Antp P1 promoter (*Antp P1*-Gal4>*SynthAntp-eGFP* long or short linker) resulted in 100% embryonic lethality, rendering the analysis of concentration and variability in imaginal discs impossible. This indicated that an indiscriminate increase of the dose of either Antp variant from early embryonic development onwards cannot be tolerated or buffered by the auto-regulatory circuit.

**Fig. 6.**
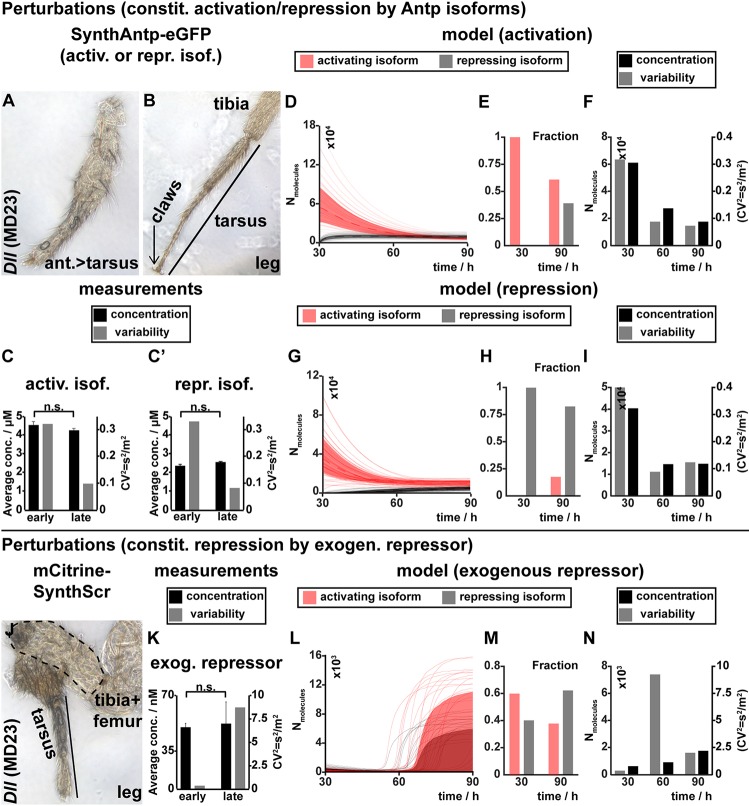
**Response of *Antp* to genetic perturbations.** (A,B) Overexpression of *SynthAntp-eGFP* long or short linker isoform result in tarsal transformations of the antenna (A), but normal leg development (B). These flies are fully viable and can be maintained as a stock. (C,C′) Antp concentration and variability, measured by FCS, in leg discs of second and third instar larvae upon SynthAntp-eGFP long or short linker isoform expression. Despite a persistent high concentration of Antp due to overexpression, variability is reduced. (D-I) Model response upon overexpression of Antp-activating or -repressing isoforms (similar to Fig. 5H-J). Trajectories of individual simulations are presented in D and G and the distribution of the *Antp* isoforms, predicted by the model, are presented in E and H. (J) Overexpression of an exogenous repressor (*Scr*) results in abnormal distal leg development, bearing malformations of the tarsus and femur. (K-N) Similar to C-I (see also Fig. S14E-I′). Antp concentration and variability, measured by FCS in the proximal leg disc of second (early) and third (late) instar larvae upon overexpression of mCherry-SynthScr.

However, overexpression from a *Dll* enhancer [*Dll*-Gal4 (MD23)] in the leg discs or in the notum (*MS243*-Gal4), which overlaps with the endogenous Antp expression pattern only during first instar disc development (Emerald and Cohen, 2004), resulted in normal adult leg and notum structures. Flies overexpressing either the *SynthAntp* auto-activating or the auto-repressing isoform in distal appendages (Fig. 6A,B) or the notum (Fig. S14A) displayed the wild-type morphology, indicative of normal Antp function, regardless of which isoform (activating or repressing) was overexpressed. We further measured by FCS the concentration and variability of the total Antp protein (endogenous Antp-eGFP and overexpressed SynthAntp-eGFP) in proximal regions of the leg disc at second and third instar stages (Fig. 6C,C′). We found that the concentration remained high at both stages due to overexpression, but variability was reduced to endogenous levels at late stages. In addition, the reduced Antp variability does not seem to depend on Antp concentration alone, because for high concentrations at both early and late stages, variability is high only in the early stage and reduced in the late stage. Together, the phenotypic analysis and FCS measurements indicate that *Antp* auto-regulation is able to reduce variability, even at high levels of expression of either isoform, ensuring proper leg development.

The experimental data were corroborated by the model, which predicted that more than threefold overexpression of either auto-activating or auto-repressing Antp isoforms (Fig. 6E,H) will nevertheless equilibrate to normal expression levels at later stages (Fig. 6D,F,G,I). Specifically, we have measured by FCS roughly 15,400 molecules in the wild-type leg disc, and the model is in good quantitative agreement with this measurement upon overexpression of the activating or repressing isoform. In addition, there is no negative effect on the noise-suppressing property of the circuit (Fig. 6F,I). Thus, both the model and experimental data indicate that transient high levels of either isoform early during disc development can be tolerated and that the concentration and cell-to-cell variability of the endogenous Antp protein is restored at later stages.

In contrast, overexpression of an exogenous repressor, such as Sex combs reduced (Scr), which can repress Antp at the transcriptional level, but can neither activate it nor activate its own transcription (Fig. S14E-J′), resulted in abnormal leg (Fig. 6J) and notum (Fig. S14B) development. These flies died as pharate adults with malformed legs, in line with Antp being required for proper leg development in all ventral thoracic discs (legs). FCS measurements in the corresponding proximal leg disc cell nuclei of second and third instar leg discs overexpressing *mCherry-SynthScr* revealed pronounced reduction in Antp concentration and remarkable increase in variability (Fig. 6K). In agreement, the model predicted a similar block of transcription and correspondingly severe effects on Antp dynamics (Fig. 6L-N). In both the measurements and the model prediction, the high increase in variability was triggered by the fact that a majority of the cells do not ‘manage’ to switch into the highly-expressing state, as too few long-linker Antp molecules are present to establish the positive auto-regulation. Because splicing favors the short-linker isoforms at later stages, these cells never ‘recover’ from Scr repression after restriction of the Antp overexpression domain to proximal regions of the leg disc (Fig. 6L). Taken together, the minimal model of Antp auto-regulatory genetic circuit is able to explain the experimentally observed differences in *Antp* concentration and cell-to-cell variability at early and late developmental stages.

## DISCUSSION

In this work, we found that *Antp* auto-regulates its expression levels during development, starting from a preferentially auto-activating state early and transitioning to a preferentially auto-repressing state later. The early state is characterized by lower average Antp concentrations and high variability, whereas the opposite is true for the later repressing state. Without excluding other mechanisms, such as chromatin configuration, accessibility of Hox binding sites to Antp, the differential abundance of co-factors among developmental stages or different modes of interactions with different Antp isoforms, we have shown that differential expression of Antp isoforms is one contributing mechanism for the observed regulatory switch. These isoforms have preferentially activating or repressing activities on the *Antp* promoter, bind chromatin with different affinities and are themselves expressed in different relative amounts during development. A loss-of-function analysis of the isoforms *in vivo* will be required to provide a definitive answer on the relative contribution of the Antp isoform-mediated auto-regulatory circuit towards the observed suppression of variability. CRISPR/Cas9-mediated genome manipulation, in principle, allows the generation of Antp loci that express only one or the other isoform. However, it is not clear whether these flies can reach the larval developmental stages, given the Antp embryonic functions and, in fact, strong biases towards only the activating or repressing isoform introduced by *Antp*-Gal4-mediated expression of either Antp isoform resulted in embryonic lethality. In the absence of such direct evidence, we turned to mathematical modelling and derived, based on our experimental data, a simple kinetic model of *Antp* auto-regulation that confirmed the plausibility of the proposed mechanism. In addition, the model generated predictions that could be verified by introducing genetic perturbations.

Negative auto-regulation has been identified as a frequently deployed mechanism for the reduction of noise (cell-to-cell variability) and the increase of regulatory robustness in various systems (Becskei and Serrano, 2000; Dublanche et al., 2006; Gronlund et al., 2013; Nevozhay et al., 2009; Shimoga et al., 2013; Thattai and van Oudenaarden, 2001). Auto-repression has been described for the Hox gene *Ultrabithorax* (*Ubx*) in haltere specification and as a mechanism of controlling Ubx levels against genetic variation (Crickmore et al., 2009; Garaulet et al., 2008), as well as in *Ubx* promoter regulation in *Drosophila* S2 cells (Krasnow et al., 1989). In contrast, an auto-activating mechanism is responsible for the maintenance of *Deformed* expression in the embryo (Kuziora and McGinnis, 1988). These experiments suggest similar mechanisms for establishing (auto-activation) or limiting (auto-repression) Hox TF levels and variability in different developmental contexts.

Our data suggest that the developmental switch from auto-activation to auto-repression is, at least in part, mediated by molecularly distinct Antp linker isoforms. Differences in affinities of different Hox TF isoforms, based on their linker between the YPWM motif and the homeodomain, have also been identified for the Hox TF Ubx. Interestingly, its linker is also subject to alternative splicing at the RNA level (Reed et al., 2010). In a similar way to Antp, the long linker Ubx isoform displays 20-25% of the affinity of DNA binding, when compared with the short linker isoforms, and the two isoforms are not functionally interchangeable in *in vivo* assays. Finally, the Ubx linker also affects the strength of its interaction with the Hox co-factor Extradenticle (Exd), underscoring the functional importance of linker length in Hox TF function (Saadaoui et al., 2011).

Mathematical modeling predicts that the *Antp* auto-regulatory circuit is robust with respect to initial conditions and extrinsic noise by suppressing cell-to-cell concentration variability even at high concentrations of any of the two Antp isoforms (auto-repressing or auto-activating). This ‘buffering’ capacity on cell-to-cell variability is reflected in the ability of flies to tolerate more than threefold overexpression of *Antp* without dramatic changes in endogenous Antp levels or generation of abnormal phenotypes. Therefore, two different isoforms produced from the same gene with opposing roles in transcriptional regulation and different auto-regulatory binding sites on the promoter of the gene seem to suffice to create a robust gene expression circuit that is able to ‘buffer’ perturbations of the starting conditions.

So far, we have only been able to indiscriminately increase or decrease Antp concentration at the tissue level and record the phenotypic outcome of these boundary states. It will be interesting to test whether controlled perturbations of TF variability at the tissue level that render TF concentration patterns less, or more, noisy among neighboring cells, while maintaining similar mean protein concentrations, lead to abnormal phenotypes. The technology to selectively manipulate expression variability of specific TFs in a developing tissue is yet to be established.

## MATERIALS AND METHODS

### Fly stocks used

The Antp-eGFP MiMIC line was a kind gift from Hugo J. Bellen (HHMI - Baylor College of Medicine, Duncan Neurological Research Institute Houston, TX, USA; Bloomington Drosophila Stock Center, 59790). The *atonal* (VDRC ID 318959), *brinker* (VDRC ID 318246), *spalt major* (VDRC ID 318068), *yorkie* (VDRC ID 318237), *senseless* (VDRC ID 318017) and *Sex combs reduced* (VDRC ID 318441) fosmid lines are available from the Vienna Drosophila Resource Center (VDRC) and were generated recently in our laboratory (Sarov et al., 2016). The *fork head* (stock 43951), *grainy head* (stock 42272), *Abdominal B* (stock 38625), *eyeless,* (stock 42271), *spineless* (transcript variant A, stock 42289) and *grain* (stock 58483) tagged BACs were generated by Rebecca Spokony and Kevin P. White (Department of Human Genetics, The University of Chicago, IL, USA) and are available at the Bloomington Stock Center. For the *scalloped* gene, a GFP-trap line was used (Buszczak et al., 2007), a kind gift from Allan C. Spradling's laboratory (line CA07575), with which genome-wide chromatin immunoprecipitation experiments have been performed (Slattery et al., 2013). For the *spineless* gene, Bloomington stock 42676, which tags isoforms C and D of the Spineless protein, has been also tried in fluorescence imaging and FCS experiments, but did not yield detectable fluorescence in the antennal disc, rendering it inappropriate for use in our analysis. Therefore, we used stock 42289, which tags the A isoform of theprotein. For the *eyeless* gene, the FlyFos015860(pRedFlp-Hgr)(ey13630::2XTY1-SGFP-V5-preTEV-BLRP-3XFLAG)dFRT line (VDRC ID 318018) has been tried also in fluorescence imaging and FCS experiments, but did not yield detectable fluorescence in the eye disc for it to be used in our analysis. The *act5C*-FRT-yellow-FRT-Gal4 (*Ay*-Gal4) line used for clonal overexpression or RNAi knockdown has been described (Ito et al., 1997). The UAS-*Antp* lines (synthetic and full-length), as well as UAS-*SynthScr* constructs, have been previously described (Papadopoulos et al., 2011, 2010). The *Dll*-Gal4 (MD23) line was a kind gift from Ginés Morata (CBMSO, Universidad Autónoma de Madrid, Spain) (Calleja et al., 1996). *69B*-Gal4 and *ptc*-Gal4 were obtained from the Bloomington Stock Center. The *Antp* P1-*lacZ* and P2-*lacZ* have been previously described (Engstrom et al., 1992; Zink et al., 1991). The P1 reporter construct spans the region between 9.4 kb upstream of the P1 promoter transcription initiation site and 7.8 kb downstream into the first intron, including the first exon sequences and thus comprising 17.2 kb of Antp regulatory sequences (pAPT 1.8). The line used was an insertion of the pAPT 1.8 vector bearing the P1 promoter regulatory sequences upstream of an *actin*-*lacZ* cytoplasmic reporter and was inserted in cytogenetic location 99F on the right chromosomal arm of chromosome 3. The *Antp*-RNAi line was from VDRC, line KK101774. UAS-*eGFP* stock was a kind gift from Konrad Basler (Universität Zürich, Switzerland). We are indebted to Sebastian Dunst (Max-Planck Institute of Molecular Cell Biology and Genetics, Dresden, Germany) for generating the *ubi*-FRT-mCherry(stop)-FRT-Gal4(VK37)/CyO line, which drives clonal overexpression upon flippase excision, while simultaneously marking cells by the loss of mCherry. For red-color labeling of clones the *act5C*-FRT-CD2-FRT-Gal4, UAS-*mRFP1* (NLS)/TM3 stock 30558 from the Bloomington Stock Center was used. For marking the ectopic expression domain of untagged Antp proteins, the UAS-*mRFP1*(NLS)/TM3 stock 31417 from the Bloomington Stock Center was used. The *MS243*-Gal4; UAS-*GFP*/CyO line was a kind gift from the laboratory of Ernesto Sánchez-Herrero (CBMSO, Universidad Autónoma de Madrid, Spain).

### Fly genotypes corresponding to fluorescence images

Fig. 2B,B′: *hs*-flp/+; *act5C*-FRT-yellow-FRT-Gal4/+; UAS-SynthAntp long linker-eGFP/+

Fig. 2C,C′: *hs*-flp/+; *act5C*-FRT-yellow-FRT-Gal4, UAS-eGFP/+; UAS-Antp long linker (full-length, untagged)/+

Fig. 2G,G′: *Dll*-Gal4 (MD23)/+; UAS-SynthAntp-eGFP/*Antp* P1-*lacZ*

Fig. 3B,B′,G,G′: *hs*-flp/+; *ubi*-FRT-mChery-FRT-Gal4/+; Antp-eGFP (MiMIC)/UAS-Antp long linker (full-length, untagged)

Fig. 3C,C′: *hs*-flp/+; UAS-Antp*^RNAi^*/+; *Antp* P1-*lacZ*/*act5C*-FRT-CD2-FRT-Gal4, UAS-mRFP1(NLS)

Fig. 3H,H′: *hs*-flp/+; UAS-Antp*^RNAi^*/*act5C*-FRT-yellow-FRT-Gal4, UAS-eGFP; *Antp* P1-*lacZ*/+

Fig. 4B,B′: *ptc*-Gal4/+; UAS-SynthAntp long linker-eGFP/+

Fig. 4C,C′: *Dll*-Gal4 (MD23)/+; UAS-SynthAntp long linker-eGFP/*Antp* P1-*lacZ*

Fig. 4F,F′: *ptc*-Gal4/+; UAS-SynthAntp long linker-eGFP/+

Fig. 4G,G′: *Dll*-Gal4 (MD23)/+; UAS-SynthAntp short linker-eGFP/*Antp* P1-*lacZ*

Fig. 5A: *Dll*-Gal4 (MD23)/+; UAS-SynthAntp long linker-eGFP/+

Fig. 5B: *ptc*-Gal4/+; UAS-SynthAntp long linker-eGFP/+

Fig. 5C: *Dll*-Gal4 (MD713)/+; UAS-SynthAntp long linker-eGFP/+

Fig. 5D: *69B*-Gal4/UAS-SynthAntp long linker-eGFP

Fig. 6A,B: *Dll*-Gal4 (MD23)/+; UAS-SynthAntp long linker-eGFP/+ or *Dll*-Gal4 (MD23)/+; UAS-SynthAntp short linker-eGFP/+

Fig. 6J: *Dll*-Gal4 (MD23)/+; UAS-mCitrine-SynthScr/+

### Preparation of second and third instar imaginal discs for FCS measurements

For FCS measurements, imaginal discs (eye-antennal, wing, leg, humeral and genital) and salivary glands were dissected from third instar wandering larvae, or wing and leg discs from second instar larvae, in Grace's insect tissue culture medium (Thermo Fisher Scientific, 11595030) and transferred to an eight-well chambered coverglass (Nunc Lab-Tek, 155411) containing PBS just prior to imaging or FCS measurements. Floating imaginal discs or salivary glands were sunk to the bottom of the well using forceps.

### Immunostainings in larval imaginal discs

Larval imaginal discs were stained according to Papadopoulos et al. (2010). Staining for the endogenous Antp protein were performed using a mouse anti-Antp antibody (Developmental Studies Hybridoma Bank, University of Iowa, anti-Antp 4C3) in a dilution of 1:250 for embryos and 1:500 for imaginal discs. eGFP, or eGFP-tagged proteins, were stained using mouse or rabbit anti-GFP antibodies from Thermo Fisher Scientific at 1:500 in imaginal discs and 1:250 in embryos. mRFP1 was stained using a Chromotek rat anti-RFP antibody. For *Antp* P1 promoter staining in imaginal discs, we used the mouse anti-β-galactosidase 40-1a antibody from Developmental Studies Hybridoma Bank, University of Iowa at 1:50. The rabbit anti-Scr antibody was used at 1:300 (LeMotte et al., 1989). Confocal images of antibody staining represent predominantly *z*-projections and Zeiss LSM510, Zeiss LSM700 or Zeiss LSM880 Airyscan confocal laser-scanning microscopy systems with an inverted stand Axio Observer microscope were used for imaging. Image processing and quantifications have been performed in Fiji (Schindelin et al., 2012). For optimal spectral separation, secondary antibodies coupled to Alexa405, Alexa488, Alexa594 and Cy5 (Thermo Fisher Scientific) were used.

### Colocalization of wild-type and eGFP-tagged MiMIC *Antp* alleles in imaginal discs

To examine whether the pattern of the MiMIC Antp-eGFP fusion protein recapitulates the *Antp* wild-type expression pattern in both embryo and larval imaginal discs, we performed immunostaining of heterozygous *Antp-eGFP* and wild-type flies to visualize the embryonic (stage 13) and larval expression of *Antp* and *eGFP*. In this experiment, we (1) visualized the overlap between eGFP and *Antp* (the eGFP pattern reflects the protein encoded by the MiMIC allele, whereas the *Antp* pattern reflects the sum of protein produced by the MiMIC allele and the allele of the balancer chromosome); and (2) compared the eGFP expression pattern to the Antp expression pattern in wild-type discs and embryos.

### Induction of early and late overexpression and RNAi-knockdown clones in imaginal discs

Genetic crosses with ∼100 virgin female and 100 male flies were set up in bottles and the flies were allowed to mate for 2 days. Then, they were transferred to new bottles and embryos were collected for 6 h at 25°C. Flies were then transferred to fresh bottles and kept until the next collection at 18°C. To asses Antp auto-activation, the collected eggs were allowed to grow at 25°C for 26 h from the midpoint of collection, when they were subjected to heat-shock by submersion of the bottles in a water bath at 38°C for 30 min and then placed back at 25°C until they reached the stage of third instar wandering larvae, when they were collected for dissection, fixation and staining with antibodies. To assess Antp auto-repression, the same procedure was followed, except that the heat-shock was performed at 60 h of development after the midpoint of embryo collection. Whenever necessary, larval genotypes were selected under a dissection stereomicroscope with green and red fluorescence filters on the basis of *deformed* (*dfd*)-YFP bearing balancer chromosomes (Le et al., 2006) and visual inspection of fluorescence in imaginal discs.

### Measurement of Antp transcript variant abundance

The linker between the Antp YPWM motif and the homeodomain contains the sequence RSQFGKCQE. Short linker isoforms encode the sequence RSQFE, whereas long linker isoforms are generated by alternative splicing of a 12 base pair sequence encoding the four amino acid sequence GKCQ into the mRNA. We initially designed primer pairs for RT-qPCR experiments to distinguish between the short and long linker mRNA variants. For the short linker variant, we used nucleotide sequences corresponding to RSQFERKR (with RKR being the first 3 amino acids of the homeodomain). For detection of the long linker variant, we designed primers either corresponding to the RSQFGKCQ sequence or to GKCQERKR. We observed in control PCRs (using plasmid DNA harboring either a long or a short linker cDNA) that primers designed for the short linker variant still amplified the long linker one. Moreover, with linker sequences differing in only four amino acids, encoded by 12 base pairs, primer pairs flanking the linker could also not be used, because, owing to very similar sizes, both variants would be amplified in RT-qPCR experiments with almost equal efficiencies. Therefore, we used primer pairs flanking the linker region to indiscriminately amplify short and long linker variants, using non-saturating PCR (18 cycles) on total cDNA generated from total RNA. We then resolved and assessed the relative amounts of long and short linker amplicons in a second step using Fragment Analyzer (Advanced Analytical). RNA was extracted from stage 13 embryos, second instar larvae at 60 h of development, and leg or wing discs from third instar wandering larvae using the Trizol reagent (Thermo Fisher Scientific), following the manufacturer's instructions. Total RNA amounts were measured using NanoDrop and equal amounts were used to synthesize cDNA using the High-Capacity RNA-to-cDNA Kit (Thermo Fisher Scientific), following the manufacturer's instructions. Total cDNA yields were measured by NanoDrop and equal amounts were used in PCR, using in-house produced Taq polymerase. Plasmid DNA (10 ng), bearing either a long or a short transcript cDNA were used as a control. PCR product abundance was analyzed both by agarose gel electrophoresis and using Fragment Analyzer.

The quantification of the transcript variant concentration (Fig. 4D,H) was made by considering 100% (value equal to 1 on the *y*-axis) as the sum of long and short isoforms at each developmental stage, whereas the quantification of the relative activation and repression efficiency was performed considering the short linker variant as having 100% repression and the long linker variant as having 100% activation efficiency (values equal to 1 on the *y*-axis).

### Quantification of the relative repressing and activating efficiencies of different Antp isoforms

Quantification of the relative efficiency of Antp activating and repressing isoforms (Fig. 4D,H) were performed in Fiji (Schindelin et al., 2012) by outlining the total region of repression or activation of Antp protein or P1 reporter staining and quantification of the relative fluorescence intensity of the selected regions. From the calculated values, we have subtracted the values obtained by outlining and calculating Antp protein or reporter β-galactosidase staining background in the region of expression of an eGFP-encoding transgene alone (negative control). Five to seven imaginal disc images per investigated genotype were used for analysis. For the repression assay, the obtained values have been normalized over the intensity of Antp protein calculated in the region of overlap between an eGFP-expressing transgene and Antp (negative control). In both cases (repression and activation), the highest efficiencies per transcript variant (for repression, the short linker isoform; for activation the long linker isoform) have been set to 100%.

### Fluorescence microscopy imaging of live imaginal discs and FCS

Fluorescence imaging and FCS measurements were performed on two uniquely modified confocal laser scanning microscopy systems, both featuring the ConfoCor3 system (Zeiss) and consisting of either an inverted microscope for transmitted light and epifluorescence (Axiovert 200 M); a VIS-laser module comprising the Ar/ArKr (458, 477, 488 and 514 nm), HeNe 543 nm and HeNe 633 nm lasers and the scanning module LSM510 META; or a Zeiss LSM780 inverted setup, comprising Diode 405 nm, Ar multiline 458, 488 and 514 nm, DPSS 561 nm and HeNe 633 nm lasers. Both instruments were modified to enable detection using silicon Avalanche Photo Detectors (SPCM-AQR-1X; PerkinElmer) for imaging and FCS (Vukojevic et al., 2008). Images were recorded at a 512×512 pixel resolution. C-Apochromat 40×/1.2 W UV-VIS-IR objectives were used throughout. Fluorescence intensity fluctuations were recorded in arrays of 10 consecutive measurements, each measurement lasting 10 s. Averaged curves were analyzed using the software for online data analysis or exported and fitted offline using the OriginPro 8 data analysis software (OriginLab). In both cases, the nonlinear least square fitting of the autocorrelation curve was performed using the Levenberg**–**Marquardt algorithm. Quality of the fitting was evaluated by visual inspection and by residuals analysis. Control FCS measurements to assess the detection volume were routinely performed prior to data acquisition, using dilute solutions of known concentration of Rhodamine 6G and Alexa488 dyes. The variability between independent measurements reflects variabilities between cells, rather than imprecision of FCS measurements. For more details on fluorescence microscopy imaging and FCS, refer to the supplementary Materials and Methods.

In Fig. 1A-H the workflow of FCS measurements is schematically represented. Live imaging of imaginal discs, expressing endogenously tagged TFs, visualized by fluorescence microscopy, and neighboring cells, expressing TFs at different levels, selected for FCS measurements (Fig. 1A,B). FCS measurements were performed by focusing the laser light into the nucleus (Fig. 1C,D) and recording fluorescence intensity fluctuations (Fig. 1E), which are generated by TF molecules quickly/slowly diffusing into/out of the confocal detection volume (Fig. 1D). The recorded fluctuations are subjected to temporal autocorrelation analysis, which generates temporal autocorrelation curves (henceforth referred to as FCS curves) that, by fitting with an appropriate model function (see supplementary Materials and Methods), yield information about the absolute concentration of fluorescent molecules (Fig. 1F) and their corresponding diffusion times, as well as the fraction of fast- and slowly-diffusing TF molecules. Differences in diffusion and the fractions of faster- and slower-diffusing molecules can be readily visualized after normalization to the same amplitude (Fig. 1G). The concentration of molecules is inversely proportional to the *y*-axis amplitude at the zero lag time, i.e. the origin of the FCS curve (Fig. 1F). FCS curves normalized to the same amplitude clearly show a shift of the FCS curves to longer lag times when processes that slow down the diffusion of TF molecules, such as binding to very large molecules (e.g. chromosomal DNA), are present (Fig. 1G). Measurements in a collection of neighboring cell nuclei also allow the calculation of protein concentration variability at the live tissue level (Fig. 1H).

### Sample size, biological and technical replicates

For the measurement of TF molecular numbers and variability (Fig. 1 and Fig. S1), seven to ten larvae of each fly strain were dissected, yielding at least 15 imaginal discs, which were used in FCS analysis. For the Fkh TF, seven pairs of salivary glands were analyzed and for AbdB, 12 genital discs were dissected from 12 larvae. More than 50 FCS measurements were performed in patches of neighboring cells of these dissected discs, in the regions of expression indicated in Fig. S1 by arrows. Imaginal discs from the same fly strain (expressing a given endogenously tagged TF) were analyzed on at least three independent occasions (FCS sessions), taking place on different days (biological replicates). For Antp, which was further analyzed in this study, more than 20 independent FCS sessions were used. As routinely carried out with FCS measurements in live cells, these measurements were evaluated during acquisition and subsequent analysis, and, based on their quality (high counts per molecule and low photobleaching), were included in the calculation of concentration and variability. In Fig. S1Q, *n* denotes the number of FCS measurements included in the calculations.

For experiments involving immunostaining in imaginal discs to investigate the auto-regulatory behavior of *Antp* (Figs 2-5, except for the temporally resolved auto-activating and repressing study of Antp in Fig. 3, as discussed above), 14-20 male and female flies were mated in bottles and 10 larvae were selected by means of fluorescent balancers and processed downstream. Up to 20 imaginal discs were visualized by fluorescence microscopy and high-resolution *z*-stacks were acquired for three to five representative discs or disc regions of interest per experiment. All experiments were performed in triplicate, except for the temporal analysis of *Antp* auto-regulatory behavior in Fig. 3, which was performed six times, and the quantification of repression efficiency of short and long linker Antp isoforms in Fig. 4, which was performed five times. For the quantification of transcript variant abundance in Fig. 4D,H, RNA and, thus, cDNA were prepared from each stage three independent times (biological replicates) and the transcript abundance per RNA/cDNA sample was also analyzed three times.

For the experiments involving perturbations in *Antp* expression, during which the proper development of the leg and the notum were assessed in Fig. 5, more than 100 adult flies were analyzed and this experiment was performed more than 10 times independently.

### Statistical significance

Fig. 2D: Statistical significance was determined using a two-tailed Student's *t*-test [***P<0.001 and *P<0.05, namely *P* (repression clone vs surrounding Antp protein) = 1.36×10^−15^ and *P* (repression clone vs surrounding *Antp* reporter) = 3.17×10^−16^].

Fig. 2H: Statistical significance was determined using a two-tailed Student's *t*-test [***P<0.001 and *P<0.05, namely *P* (*Dll* expression domain vs surrounding *Antp* reporter) = 1.55×10^−17^].

Fig. 3E: Statistical significance was determined using a two-tailed Student's *t*-test {***P<0.001 and *P<0.05, namely *P* (early activation clone vs surrounding Antp protein) = 6.23×10^−13^ and *P* [early knockdown (RNAi) clone vs surrounding *Antp* reporter] = 2.98×10^−9^}.

Fig. 3F: Statistical significance was determined using a two-tailed Student's *t*-test [***P<0.001 and *P<0.05, namely *P* (2nd vs 3rd instar τ_D2_) = 7.2×10^−4^, *P* (2nd vs 3rd instar τ_D2_) = 7.2×10^−4^ and *P* (2nd vs 3rd instar variation) = 3.4×10^−2^].

Fig. 3K: Statistical significance was determined using a two-tailed Student's *t*-test {***P<0.001 and *P<0.05, namely *P* (late repression clone vs surrounding Antp protein) = 3.98×10^−17^ and *P* [late knockdown (RNAi) clone vs surrounding *Antp* reporter] = 1.16×10^−21^}.

Fig. 4D,H: Statistical significance was determined using a two-tailed Student's *t*-test between measurements performed with the long linker (auto-activating) isoform (Fig. 4D) and the short linker (auto-repressing) isoform (Fig. 4H) {***P<0.001 and *P<0.05, namely *P* (embryo long vs short concentration) = 3.16×10^−5^, *P* (2nd instar long vs short concentration) = 1.16×10^−4^, *P* (long vs short relative activation) = 4.1×10^−3^, *P* (long vs short relative activation) = 4.1×10^−3^, *P* (long vs short relative repression) = 2.4×10^−4^ and *P* [long vs short DNA-bound fraction (FCS)] = 5.6×10^−10^}.

Fig. 5E: Statistical significance was determined using a two-tailed Student's *t*-test [***P<0.001, namely *P* (*MD23* vs *ptc*) = 3.54×10^−4^ and *P* (*MD713* vs *69B*) = 4.15×10^−9^].

Fig. 6C-C′: Statistical significance was determined using a two-tailed Student's *t*-test *P* (early vs late conc. leg disc, o/e activator) = 0.679 and *P* (early vs late conc. leg disc, o/e repressor) = 0.454.

Fig. 6K: Statistical significance was determined using a two-tailed Student's *t*-test *P* (early vs late conc. leg disc, o/e exog. repr.) = 0.892.

## Supplementary Material

Supplementary information
