# Supplementary material for: Control of Hox transcription factor concentration and cell-to-cell variability by an auto-regulatory switch
Source: Development. 2019 Jan 25;146(12):dev168179. doi: 10.1242/dev.168179 (PMC6602345; doi:10.1242/dev.168179)
Supplement: Supplementary information [file develop-146-168179-s1.pdf]

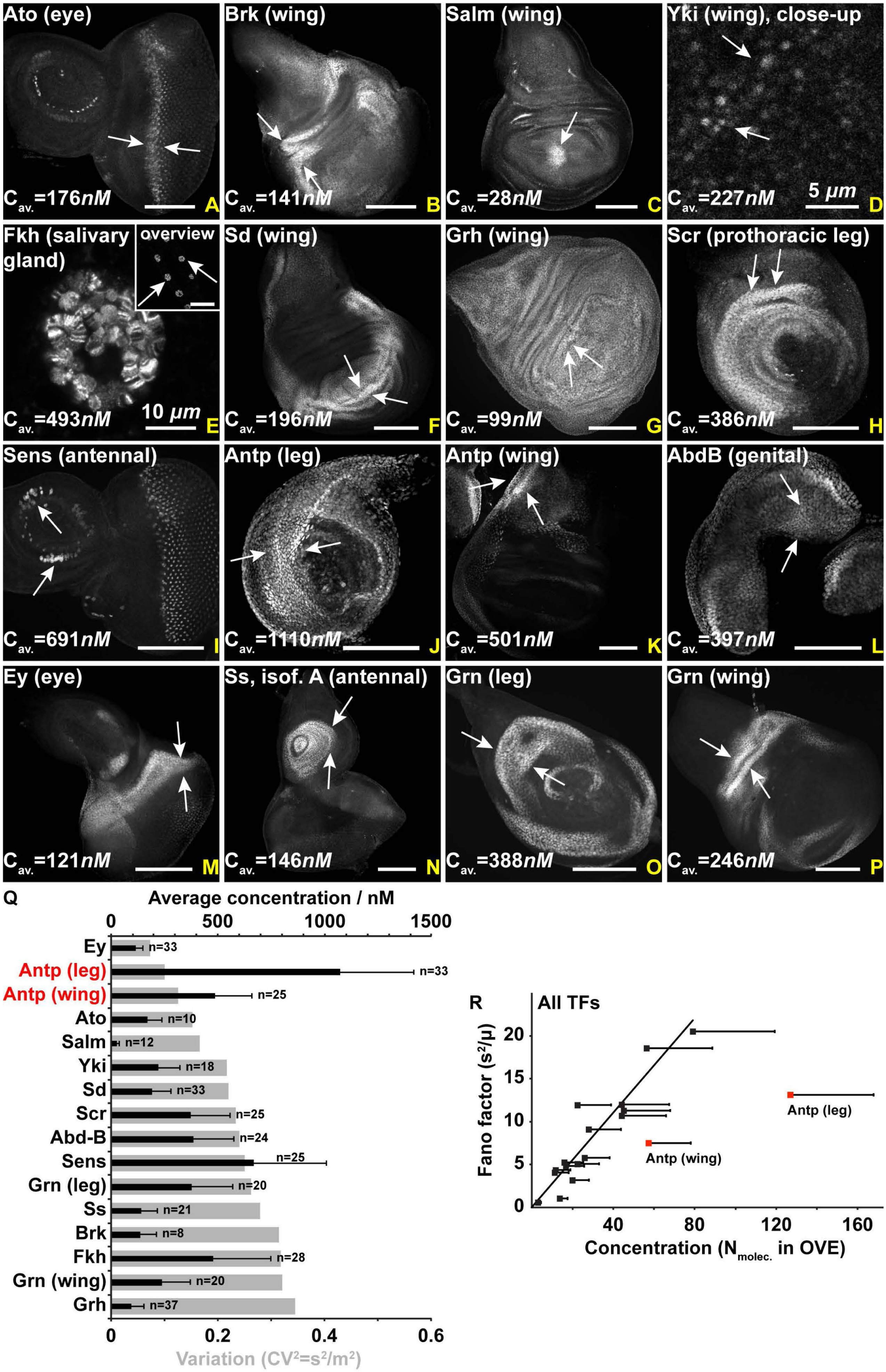

Development • Supplementary information

Figure S1. Measurement of average concentrations and nucleus-to-nucleus variability of 14 endogenously-tagged TFs in Drosophila imaginal discs by FCS. (A-P) Fluorescence imaging of TFs, showing their expression pattern in imaginal discs and the salivary gland. White arrows indicate regions where FCS measurements of endogenous intra-nuclear concentration were performed and the average concentrations are given for each TF. Images have been contrasted for visualization purposes. For the Antp and Grn TFs, both leg and wing imaginal discs have been used for measurements. Average concentrations of TFs measured in different cells span a range of two orders of magnitude, from few tens to a thousand nanomolar. Scale bars denote $100\ \mu m$, unless otherwise indicated. (Q) Characterization of nucleus-to-nucleus variability among neighboring cells within the same expression domain in imaginal discs of the 14 TF studied by FCS. Black bars show concentration averages (with error bars representing 1 standard deviation), whereas grey bars show the variability, i.e. the squared coefficient of variability (expressed as the variance over the squared mean, $CV^2 = \frac{s^2}{m^2}$). TFs have been sorted according to increasing variability. (R) Characterization of variability as a function of concentration, using the Fano factor value (expressed as variance over the mean, $F_f = \frac{s^2}{m}$). The red squares point to the F_f values of Antp in the wing and leg disc.

Development • Supplementary information

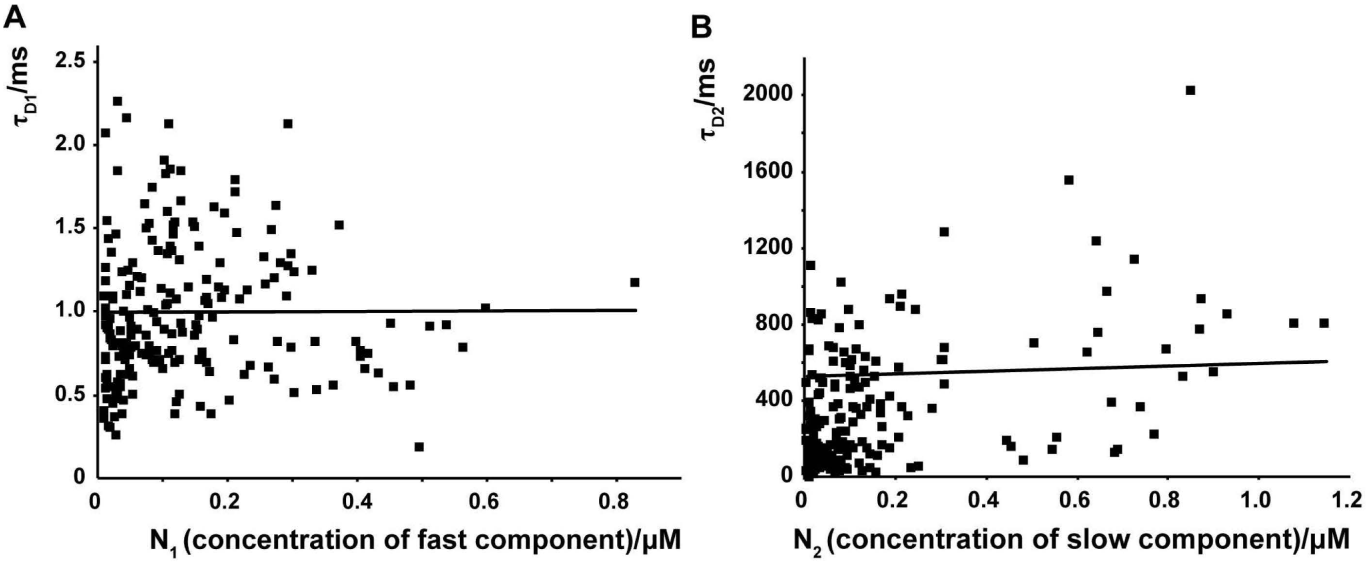

Figure S2. Characteristic decay times of Antp-eGFP do not change as a function of total concentration. (A-B) Characteristic decay times τ_{D_1} (A) and τ_{D_2} (B) do not vary with the concentration of Antp-eGFP TF molecules, as evident from $\tau_{D_1} = f(N_1)$ and $\tau_{D_2} = f(N_2)$, where N_1 is the number of freely diffusing, N_2 the number of bound Antp-eGFP TF molecules and τ_{D_1}, τ_{D_2} their respective diffusion times.

Development • Supplementary information

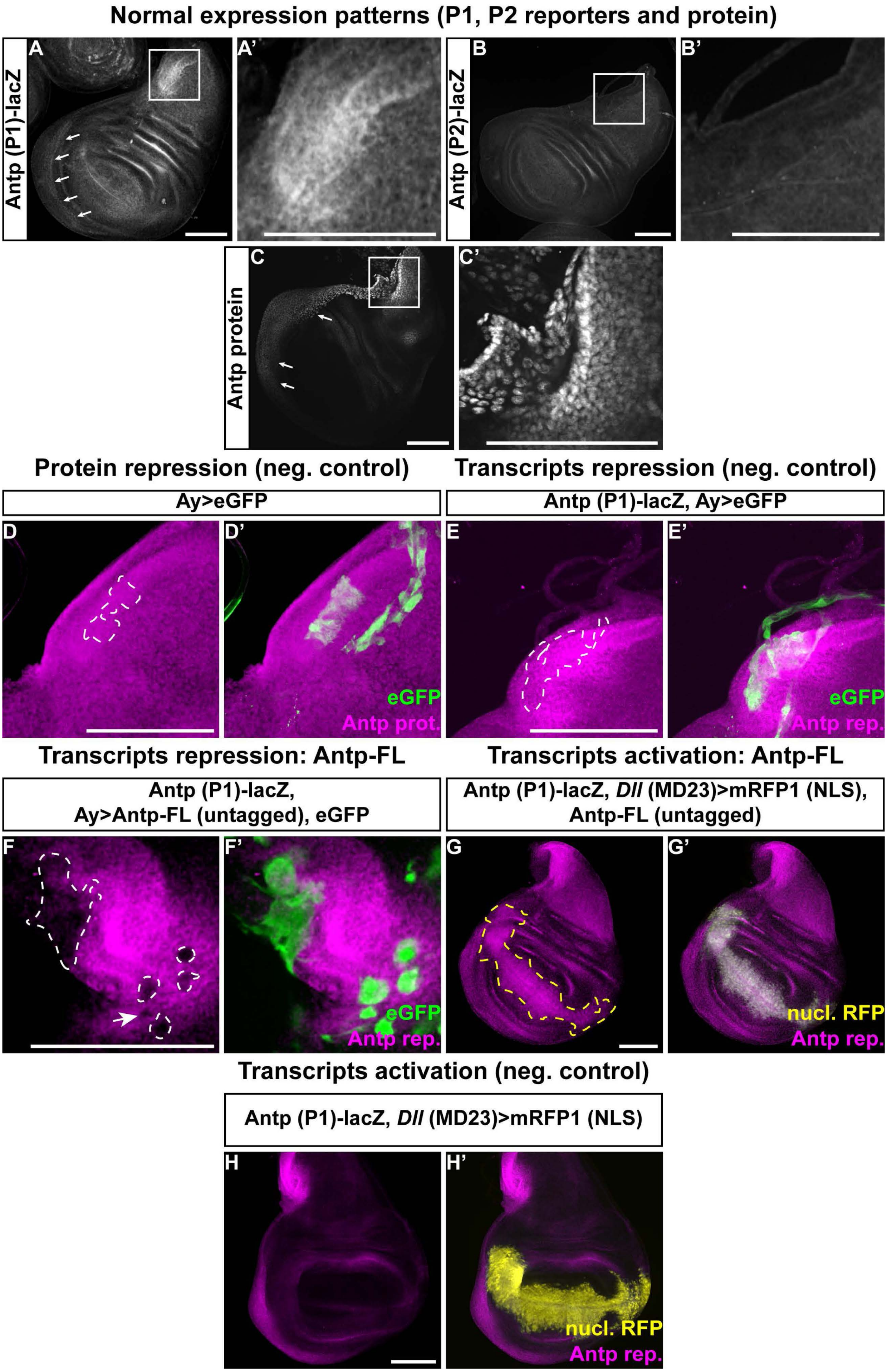
Normal expression patterns (P1, P2 reporters and protein)
Antp (P1)-lacZ
A
A'
Antp (P2)-lacZ
B
B'
Antp protein
C
C'
Protein repression (neg. control)
Transcripts repression (neg. control)
Ay>eGFP
Antp (P1)-lacZ, Ay>eGFP
D
D'
eGFP
Antp prot.
E
E'
eGFP
Antp rep.
Transcripts repression: Antp-FL
Transcripts activation: Antp-FL
Antp (P1)-lacZ,
Ay>Antp-FL (untagged), eGFP
Antp (P1)-lacZ, Dll (MD23)>mRFP1 (NLS),
Antp-FL (untagged)
F
F'
eGFP
Antp rep.
G
G'
nucl. RFP
Antp rep.
Transcripts activation (neg. control)
Antp (P1)-lacZ, Dll (MD23)>mRFP1 (NLS)
H
H'
nucl. RFP
Antp rep.

Figure S3. *Antp* is able to repress and activate itself at the transcriptional level – controls. (A-C') Normal expression patterns of the *Antp* P1 (A-A') and P2 (B-B') transcriptional reporters and Antp protein immunohistochemistry (C-C'). Boxed areas in (A), (B) and (C) are magnified in (A'), (B') and (C'). The *Antp* P1 reporter is highly expressed in the prescutum region of the notum (A') and the peripodial cells at the base of the wing blade (giving rise to the mesopleura and pteropleura of the thorax, white arrows in (A)), which overlaps with the Antp protein pattern ((C') and arrows in (C)). The *Antp* P2 promoter reporter construct exhibits very weak, if any, expression at these two domains (B-B'). (D-E') Negative controls of Antp protein (D-D') and P1 reporter transcription (E-E') upon overexpression of eGFP. Dashed lines outline the regions of clonal induction in (D) and (E), where neither the Antp protein (D) nor the *Antp* P1 reporter (E) are repressed. (F-F') Repression of *Antp* P1 reporter transcription upon clonal overexpression of the full-length untagged Antp protein (Antp-FL). The ectopic expression domain is outlined by white dashed lines in (F) and marked by the expression of eGFP (F'). (G-G') Activation of *Antp* P1 reporter transcription upon ectopic expression of untagged *Antp* full-length (*Antp*-FL) with *Dll* (MD23) driver in the distal region of wing pouch. The ectopic expression domain is outlined by a yellow dashed line in (G) and is marked by the expression of nuclear mRFP1. (H-H') Negative control of ectopic activation of *Antp* P1 transcription upon overexpression of nuclear mRFP1 alone by *Dll* (MD23)-Gal4. Scale bars denote $100\ \mu m$.

Development • Supplementary information

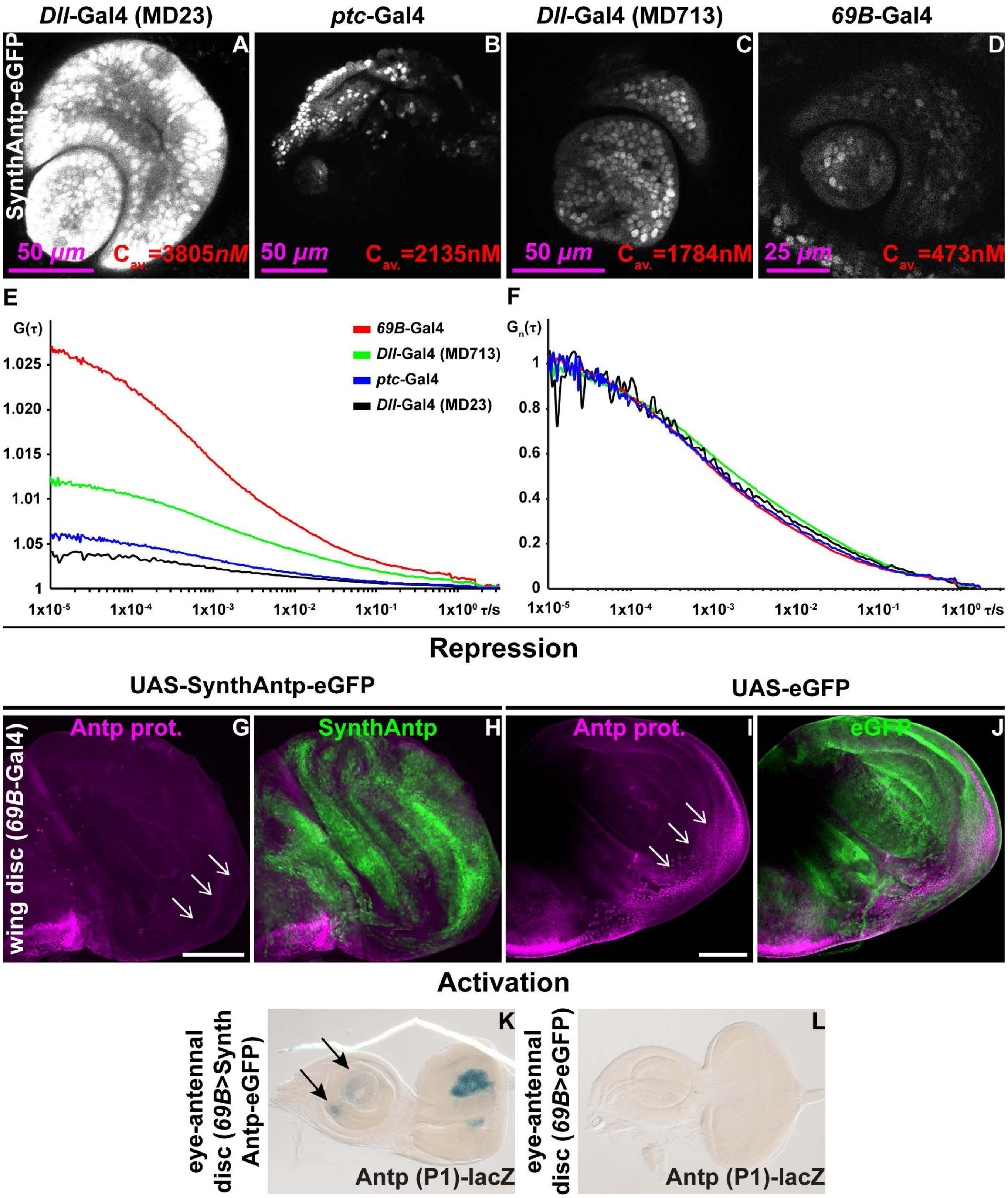

Development • Supplementary information

Figure S4. Direct correlation between Antp concentration and homeotic function – *Antp* auto-repression and activation occurs at endogenous concentrations. (A-D) Live imaging (one optical section) of *SynthAntp*-eGFP expressed in the distal antennal portion of the eye-antennal disc by different Gal4 drivers. The concentration was measured using FCS and average concentrations are indicated. An eightfold difference was observed between the strong *Dll*-Gal4 driver (MD23) (A) and weak *69B*-Gal4 driver (D). (E) Average FCS measurements performed in nuclei overexpressing *SynthAntp-eGFP*, using different Gal4 drivers. Note that the y-axis amplitudes at the origin of the FCS curves are inversely proportional to the concentration. (F) FCS curves of measurements in (E), normalized to the same amplitude, $G_n(\tau) = 1$ at $\tau = 10\ \mu s$, show major overlap, indicating indistinguishable behavior of Antp binding to chromatin across the concentration range examined ($0.5 - 3.8\ nM$). (G-L) *Antp* auto-regulation occurs at endogenous concentrations. (G-H) Repression of endogenous Antp protein upon induction of *SynthAntp-eGFP* in the proximal regions of the wing disc by *69B*-Gal4, which results in *Antp* expression very similar to endogenous levels. (I-J) No repression is observed upon overexpression of *eGFP* (negative control), as indicated by white arrows in (I). White arrows in (G) and (I) point to the equivalent area in the wing disc, where *Antp* repression is observed. (K) X-gal stainings of the *Antp* P1 reporter show weak but detectable ectopic β-galactosidase activity in the antennal disc (black arrows). (L) Negative control stainings of eGFP induced by the *69B* enhancer show complete absence of ectopic reporter transcription. Scale bars denote $100\ \mu m$, unless otherwise indicated.

Development • Supplementary information

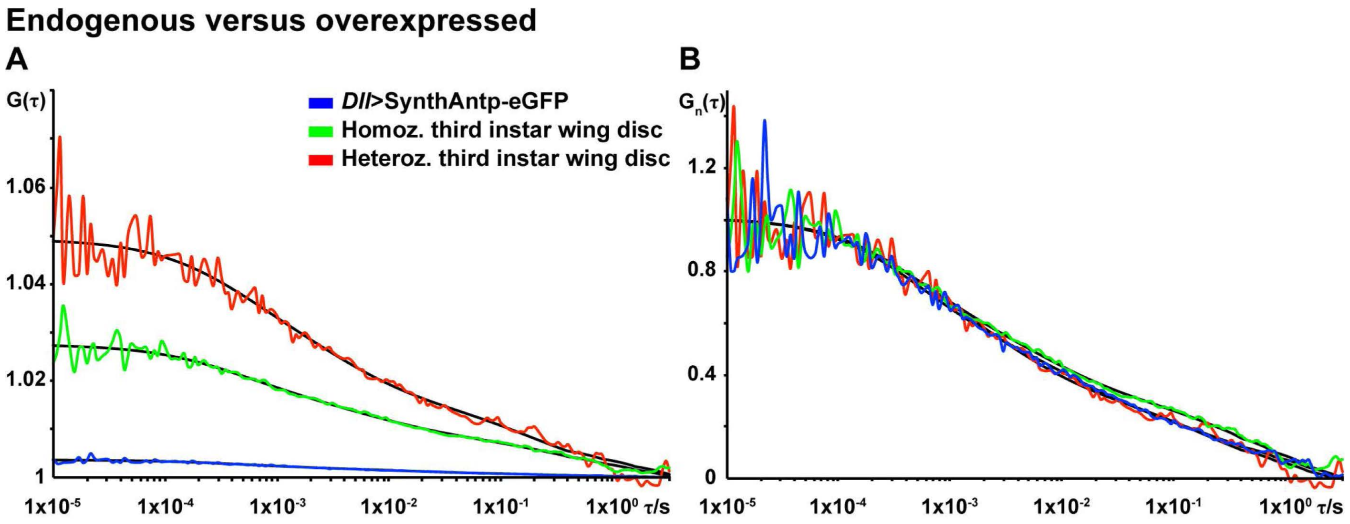

Figure S5. Comparison of endogenous and overexpressed *Antp* by FCS. (A) FCS curves of Antp-eGFP in wing disc nuclei. Concentration differences of fluorescent Antp protein are obvious among cells expressing one or two copies of *Antp-eGFP* (homozygous and heterozygous larvae) or overexpressing SynthAntp-eGFP from the *Dll* MD23)-Gal4 driver. (B) FCS curves shown in (A) normalized to the same amplitude, $G_n(\tau) = 1$ at $\tau = 10$ μs, show pronounced overlap between homozygous and heterozygous *Antp-eGFP*-expressing cells, as well as between endogenously expressed *Antp* and overexpressed *SynthAntp-eGFP*, indicating similar diffusion times and modes of interaction with chromatin. FCS curves are color-coded as outlined in panel (A).

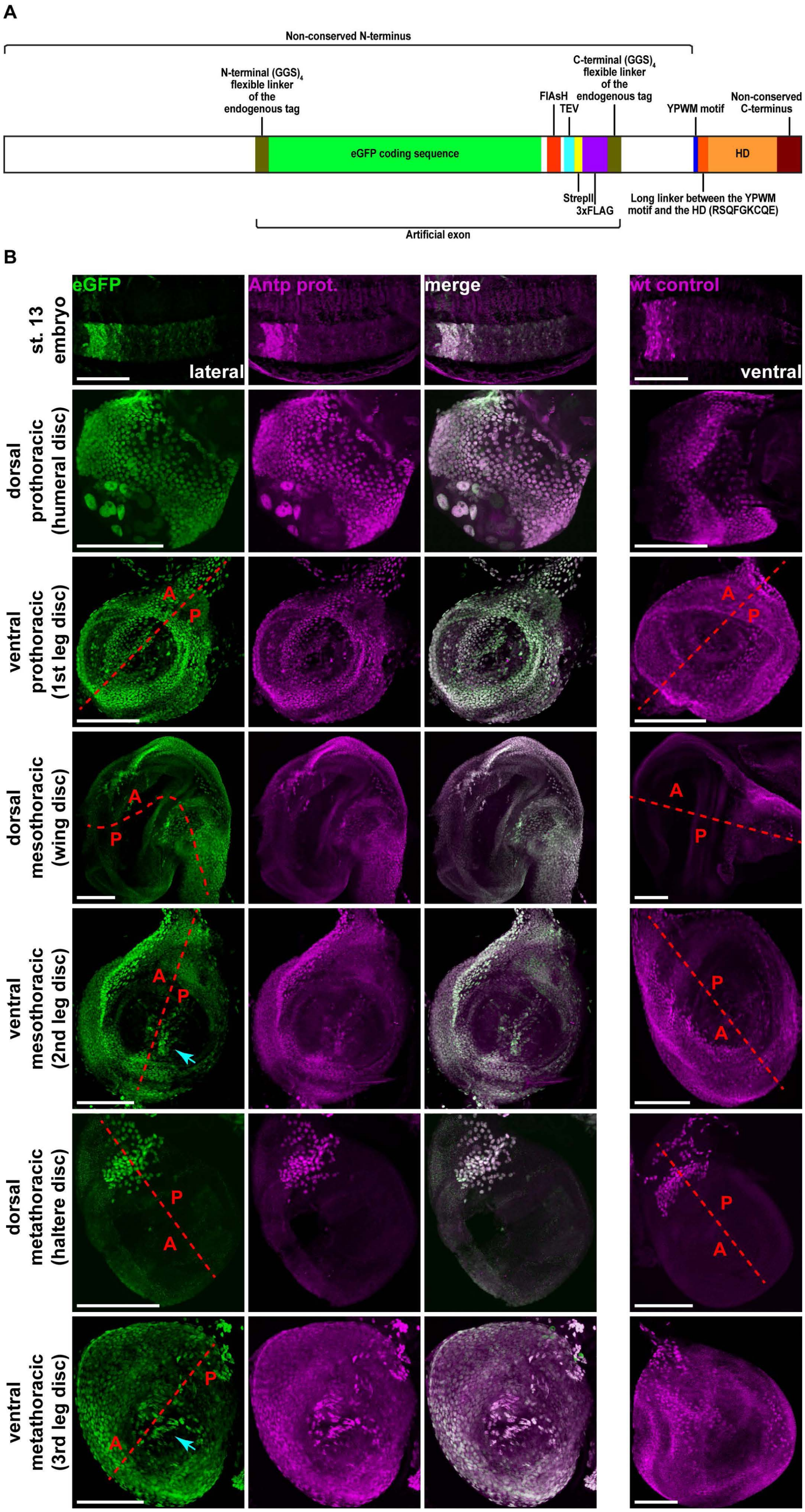

Development • Supplementary information

Figure S6. *Antp* expression patterns are not altered by the MiMIC MI02272 insertion. (A) Schematic representation of the Antp-eGFP fusion protein produced by the conversion of the MiMIC MI02272 construct to an artificial exon. The eGFP-encoding artificial exon is situated in intron 6 of the mRNA and is spliced in between exons 6 and 7 that correspond to the long and non-conserved N-terminal coding sequence of the protein, which has little (if any) function *in vivo* (Papadopoulos et al., 2011), and does not disrupt the homeodomain or YPWM motif. All features have been drawn to scale. (B) Heterozygous flies (embryos and third instar larvae), examined for their Antp-eGFP pattern (detected by an antibody to GFP, green), as compared to the total amount of Antp (expressed by the sum of the MiMIC *Antp-eGFP* and the wild type *Antp* loci), detected by an Antp antibody (magenta). Comparisons of the Antp pattern in wild type embryos and all thoracic imaginal discs are provided case-wise in the right panel. In discs, dashed lines approximately separate the anterior (indicated by “A”) from the posterior (indicated by “P”) domain of the disc. Note the high expression of *Antp* in the humeral disc. In the leg discs, Antp is expressed most strongly in the posterior compartment of the prothoracic leg disc, the anterior compartment of the mesothoracic leg disc and in an abundant pattern in the metathoracic leg disc. Cyan arrows point to Antp positive cells in the second and third leg discs that are centrally located, as previously shown (Engstrom et al., 1992). All images represent Z-projections. Scale bars denote 100 μm.

Development • Supplementary information

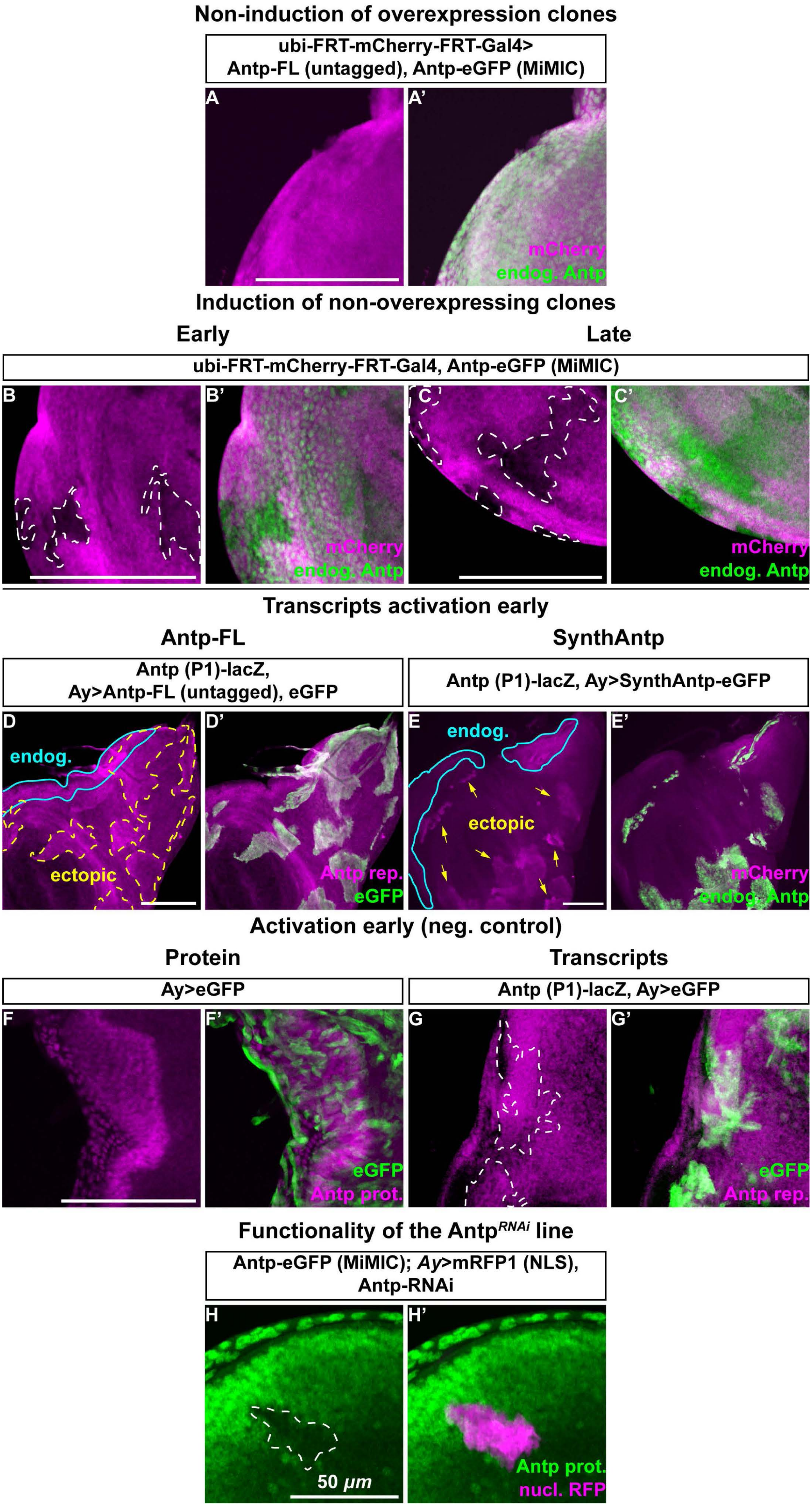

Development • Supplementary information

Figure S7. ***Antp*** **is sufficient and required to trigger a developmental switch from transcriptional auto-activation to auto-repression – controls.** (A-C') Negative controls of *Antp* clonal auto-activation and repression using early and late clone induction regimes. (A-A') Without induction of clones expressing full-length untagged *Antp*, no repression or activation of endogenous Antp protein is observed. (B-C') Upon induction of non-overexpressing clones (clones expressing only Gal4, without a UAS transgene), no activation or repression of Antp protein is observed at early or late induction time points. White dashed lines in (B) and (C) outline the induced clones, marked by the absence of mCherry. (D-E') Early ectopic induction of either Antp full-length untagged protein (D-D') or SynthAntp (E-E') result in upregulation of the Antp P1 reporter. Yellow dashed lines in (D) and arrows in (E) point to the induced clones and cyan continuous lines show the regions of high endogenous expression of the reporter. Clones have been marked by cytoplasmic eGFP. (F-G') Negative controls of early clonal induction of eGFP alone (without concurrent induction of Antp) show no repression of the Antp protein (F-F') or the P1 reporter (G-G'). Dashed lines in (G) mark the clones of eGFP induction. (H-H') Positive control of clonal knockdown of the Antp *RNAi* line used in Fig. 3. Clonal knockdown by *RNAi* (indicated by the dashed line in (H) and marked by nuclear mRFP1 in (H')) resulted in efficient downregulation of the endogenous Antp protein. Scale bars denote $100\ \mu m$, unless otherwise indicated.

Development • Supplementary information

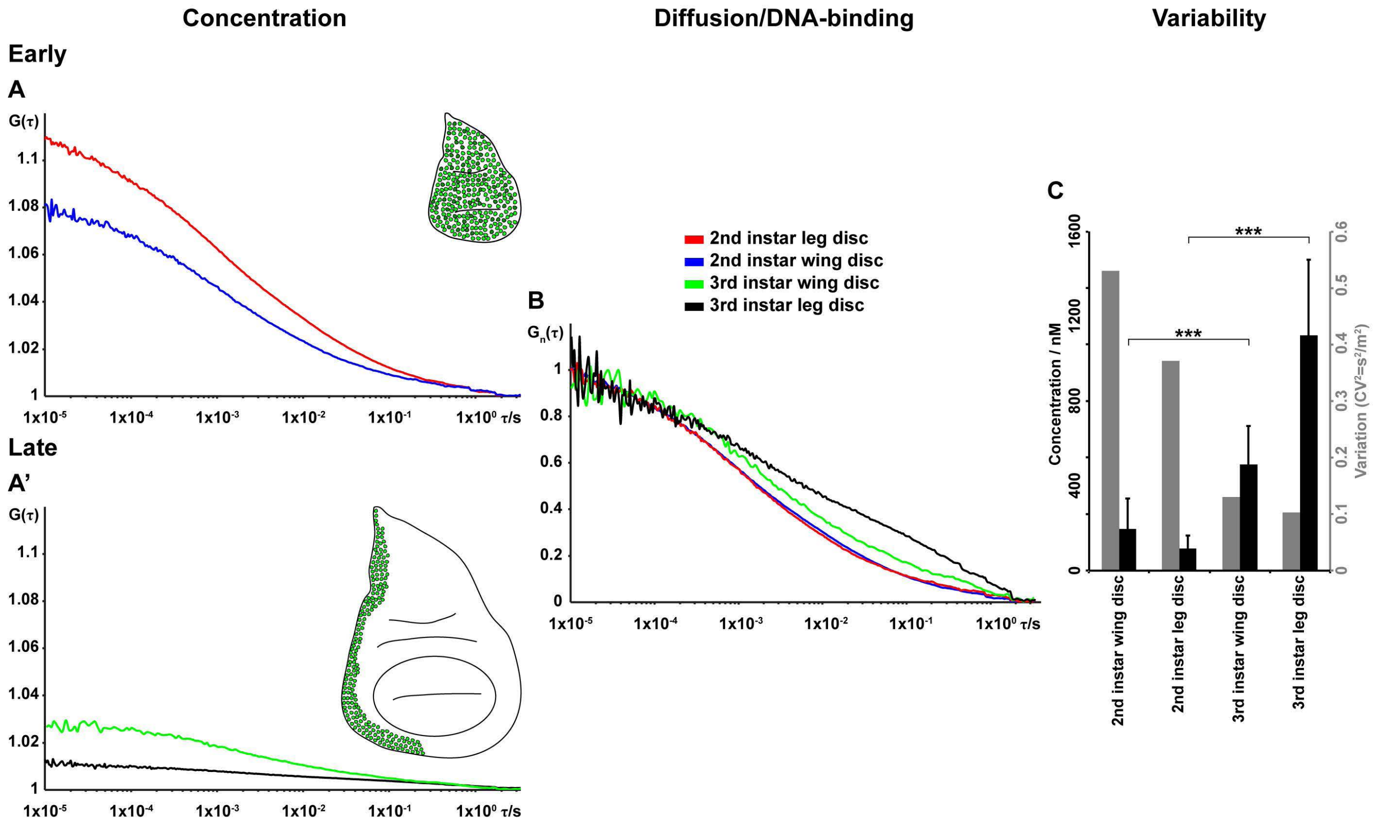

Figure S8. Antp concentration and cell-to-cell variability in second and third instar wing and leg imaginal discs (A-A'') Representative FCS curves recorded in second and third instar wing and leg imaginal discs, expressing *Antp-eGFP*. Note the low concentration in second instar leg and wing discs, reflected by the relatively high amplitude of the FCS curves (inversely proportional to concentration) in (A), as compared to the high concentration in third instar discs in (A'). (B) FCS curves shown in (A) and (A'), normalized to the same amplitude, $G_n(\tau) = 1$ at $\tau = 10\ \mu s$, show a shift towards longer decay times in the third instar leg and wing discs, indicative of pronounced interactions of Antp with chromatin. FCS curves are color-coded as outlined in panel (A). (C) Quantification of average concentrations and cell-to-cell variability in protein concentration among neighboring nuclei in wing and leg, second and third instar, discs. Black bars denote the average concentration and grey bars denote the variability, expressed as the variance over the squared mean. Note the increase in average concentration from second to third instar (eleven-fold increase in the leg disc) and the concurrent drop in variability to almost half of its value. Statistical significance was determined using Student's two-tailed T-test [****P*<0.001, namely P(3rd - 2nd instar leg) $= 4.4 \times 10^{-18}$ and *P* (3rd - 2nd instar wing)$= 3.2 \times 10^{-8}$].

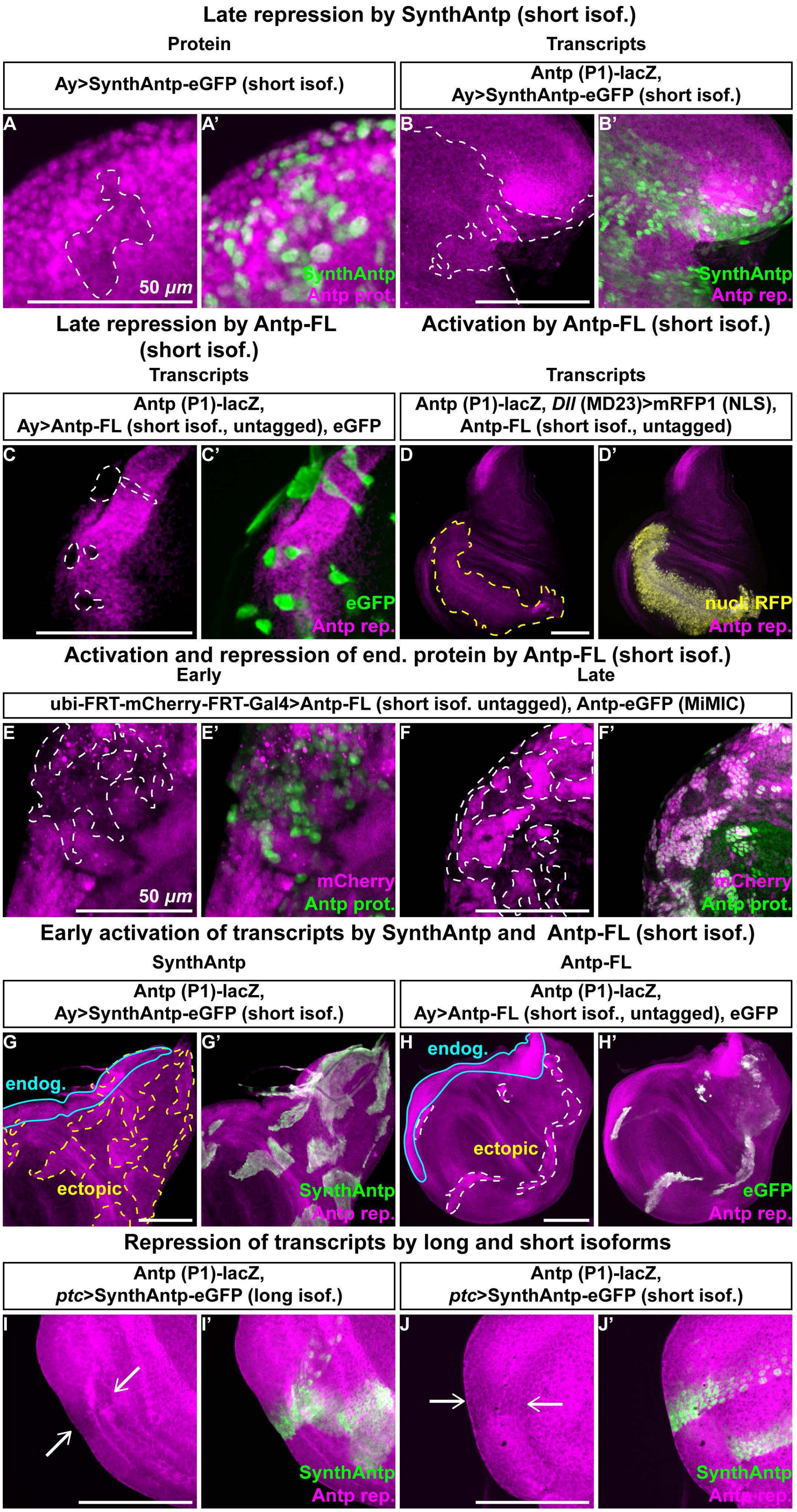
Late repression by SynthAntp (short isof.)
Protein
Transcripts
Ay>SynthAntp-eGFP (short isof.)
Antp (P1)-lacZ,
Ay>SynthAntp-eGFP (short isof.)
A
A'
B
B'
50 μm
SynthAntp
Antp prot.
SynthAntp
Antp rep.
Late repression by Antp-FL
(short isof.)
Activation by Antp-FL (short isof.)
Transcripts
Transcripts
Antp (P1)-lacZ,
Ay>Antp-FL (short isof., untagged), eGFP
Antp (P1)-lacZ, Dll (MD23)>mRFP1 (NLS),
Antp-FL (short isof., untagged)
C
C'
D
D'
eGFP
Antp rep.
nucl. RFP
Antp rep.
Activation and repression of end. protein by Antp-FL (short isof.)
Early
Late
ubi-FRT-mCherry-FRT-Gal4>Antp-FL (short isof. untagged), Antp-eGFP (MiMIC)
E
E'
F
F'
50 μm
mCherry
Antp prot.
mCherry
Antp prot.
Early activation of transcripts by SynthAntp and Antp-FL (short isof.)
SynthAntp
Antp-FL
Antp (P1)-lacZ,
Ay>SynthAntp-eGFP (short isof.)
Antp (P1)-lacZ,
Ay>Antp-FL (short isof., untagged), eGFP
G
G'
H
H'
endog.
ectopic
SynthAntp
Antp rep.
endog.
ectopic
eGFP
Antp rep.
Repression of transcripts by long and short isoforms
Antp (P1)-lacZ,
ptc>SynthAntp-eGFP (long isof.)
Antp (P1)-lacZ,
ptc>SynthAntp-eGFP (short isof.)
I
I'
J
J'
SynthAntp
Antp rep.
SynthAntp
Antp rep.

Figure S9. Developmental control of *Antp* auto-activation and repression relies on the relative concentrations of preferentially auto-activating and auto-repressing Antp isoforms, which display different binding affinities to chromatin – short linker isoform controls. (A-H') Experiments of Figs. 2-4, performed with short linker (preferentially auto-repressing) full-length and SynthAntp isoforms on their capacity to repress and activate *Antp* P1 reporter transcription and Antp protein. Dashed lines in all panels outline the clones induced or the region of ectopic expression using *Dll* (MD23)-Gal4, whereas closed continuous cyan lines outline the regions of endogenous *Antp* P1 reporter expression in (G) and (H). (A-A') Repression of Antp protein by late clonal induction of *SynthAntp* in the wing notum. (B-B') Equivalent assay as in (A-A'), but monitoring auto-repression of the *Antp* P1 promoter transcription. (C-C') Similar assay to (B-B'), using the full-length Antp protein, induced at the later time point. (D-D') Ectopic induction of full-length, short linker, untagged *Antp* cDNA with concurrent labeling of the expression domain by nuclear mRFP1 results in weak ectopic auto-activation of the *Antp* P1 reporter. (E-F') Early and late clonal induction of full-length, short linker, untagged *Antp* results in auto-repression (E-E'), or induction (F-F'), of the endogenous Antp protein, respectively. (G-H') Early clonal induction of SynthAntp (G-G') or the full-length cDNA (H-H'), both featuring a short linker, triggers ectopic activation of P1 promoter transcription. (I-J') Antp long (I-I') and short (J-J') linker isoforms repress Antp at the transcriptional level (monitored by Antp P1 reporter expression) when induced by *ptc*-Gal4 in the wing disc. Arrows point to the regions of auto-repressed *Antp* promoter. Scale bars denote 100 μm, unless otherwise indicated.

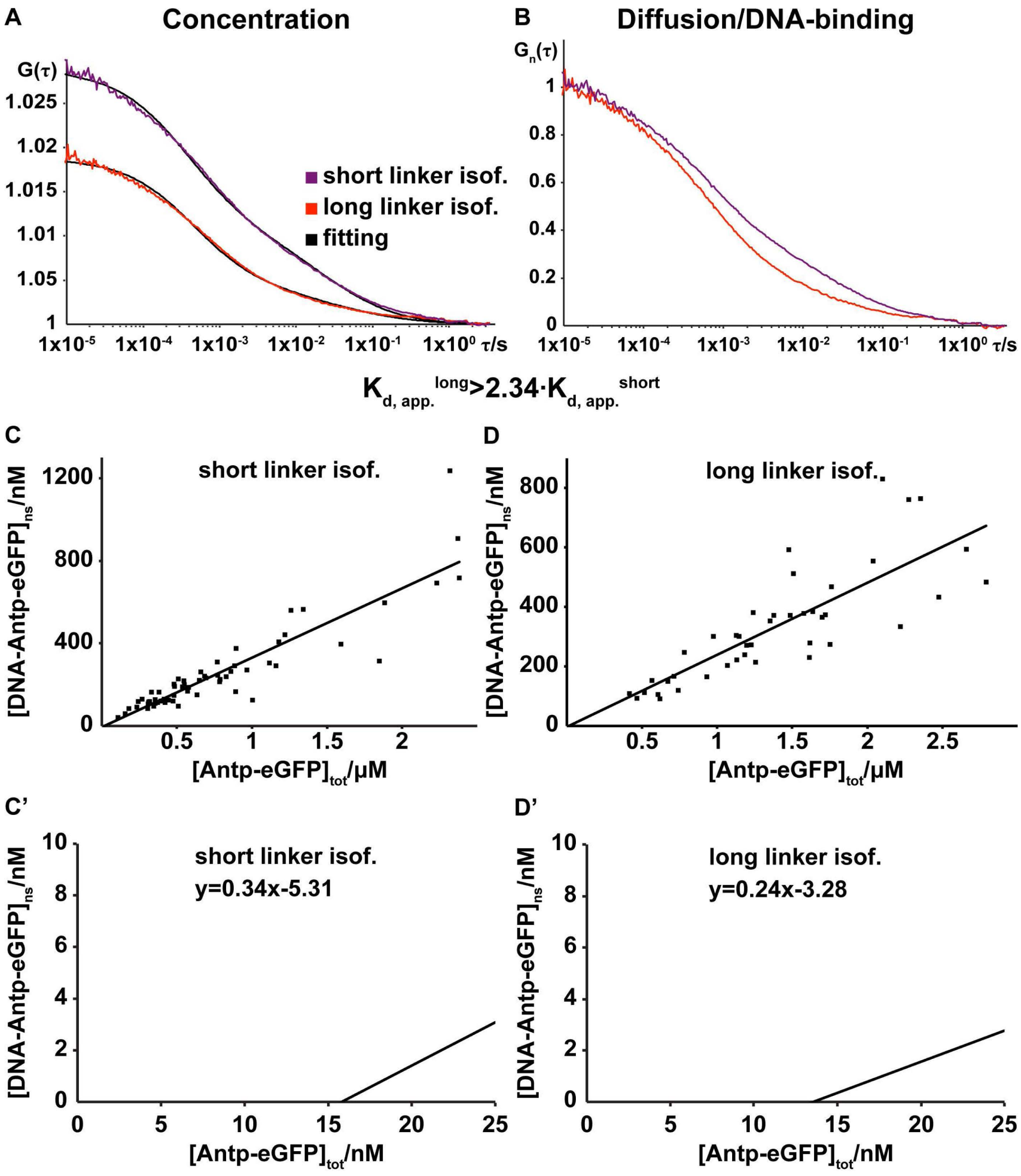
A
Concentration
G(τ)
1.025
1.02
1.015
1.01
1.05
1
1x10-5 1x10-4 1x10-3 1x10-2 1x10-1 1x10^0 τ/s
short linker isof.
long linker isof.
fitting
B
Diffusion/DNA-binding
Gn(τ)
1
0.8
0.6
0.4
0.2
0
1x10-5 1x10-4 1x10-3 1x10-2 1x10-1 1x10^0 τ/s
Kd, app.long>2.34·Kd, app.short
C
short linker isof.
[DNA-Antp-eGFP]ns/nM
1200
800
400
0
0.5 1 1.5 2
[Antp-eGFP]tot/μM
D
long linker isof.
[DNA-Antp-eGFP]ns/nM
800
600
400
200
0
0.5 1 1.5 2 2.5
[Antp-eGFP]tot/μM
C'
short linker isof.
y=0.34x-5.31
[DNA-Antp-eGFP]ns/nM
10 8 6 4 2 0
0 5 10 15 20 25
[Antp-eGFP]tot/nM
D'
long linker isof.
y=0.24x-3.28
[DNA-Antp-eGFP]ns/nM
10 8 6 4 2 0
0 5 10 15 20 25
[Antp-eGFP]tot/nM

Figure S10. Comparative binding study of Antp short and long linker isoforms by FCS. (A-B) FCS analysis performed on third instar wing and antennal imaginal discs, expressing short or long linker Antp isoforms (tagged to eGFP) close to endogenous concentrations, from the *69B*-enhancer. Cell nuclei of similar concentrations in the two datasets have been selected for analysis (A). Average FCS measurements on the short linker Antp isoform display a consistent shift towards longer decay times, as compared to its long linker counterpart (B), indicating higher degree of chromatin binding. (C-D') Binding study of short and long linker Antp isoforms in third instar wing and antennal discs, expressed by *69B*-Gal4. The concentration of the Antp short and long linker isoform DNA-bound complexes (derived by fitting the FCS curves in (A)) is plotted as a function of the total concentration of Antp-eGFP molecules. From the linear regression equations, $y = 0.34x - 5.31$ (D') and $y = 0.24x - 3.28$ (E'), the ratio of apparent dissociation constants for the long and short linker isoforms was calculated to be $\frac{K_{d,\,Antp\ long\ linker\ isof.}}{K_{d,\,Antp\ short\ linker\ isof.}} > 2.3$ (for the calculation refer to Supplement 3). The two dissociation constants differ at least 2.3 times, indicating stronger binding of the short linker isoform to the DNA, as compared to the long linker one.

Development • Supplementary information

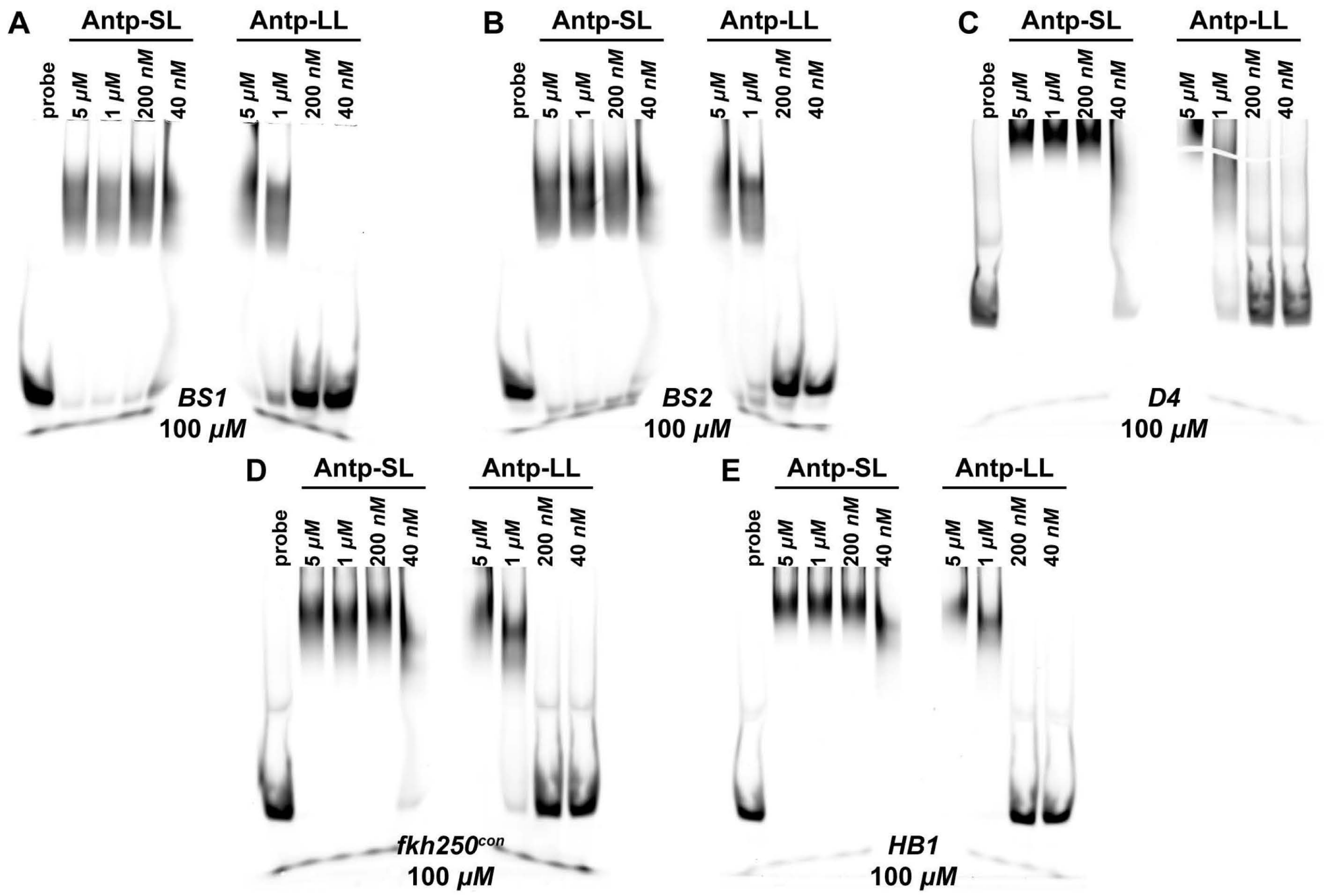

Figure S11. *In vitro* binding study of Antp full-length long and short linker isoforms to Antp and homeodomain binding sites by gel-shift assays (Electrophoretic Mobility Shift Assays – EMSAs). Full-length *Antp* short and long linker variants (transcript variants RM and RN), encoding activating and repressing Antp isoforms, respectively, were cloned into the pET21b(+) vector (Novagen), which features a C-terminal 6xHis tag, and expressed in Rosetta^{TM} 2 cells (Novagen), following the manufacturer's standard protocol. The two proteins were then Ni-column purified and subjected to gel-filtration. The concentrations of purified proteins were then compared by Western blotting, using the anti-Antp 4C3 antibody (Developmental Studies Hybridoma Bank, University of Iowa), and equal starting concentrations were used in the indicated serial dilutions (A-E) in gel-shift experiments. The BS1and BS2 binding sites have been identified ~2 kb upstream of the *engrailed* gene promoter and characterized for Antp binding previously (Affolter et al., 1990). The HB1 binding site has been described previously (Keegan et al., 1997) and is a binding site found in the intron of the mouse *Hoxa-4* gene. The D4 probe has been characterized previously (Duncan et al., 2010) as a functional element in the *spineless* gene. The *fkh250con* binding site has been described previously (Ryoo and Mann, 1999). The same procedure was followed for EMSA, as previously described (Bhatia et al., 2013). Double-stranded DNA fragments were purchased from Integrated DNA Technologies and were 5' 6-FAM end-labelled. Images were obtained using a Fujifilm FLA-5100 Fluorescent Image Analyser. (A-E) Gel-shift experiments using purified full-length Antp protein, featuring a long or a short linker, with 100 μM fluorescently labelled probe show stronger binding of the short linker isoform to all investigated binding sites, as compared to its long linker counterpart.

Development • Supplementary information

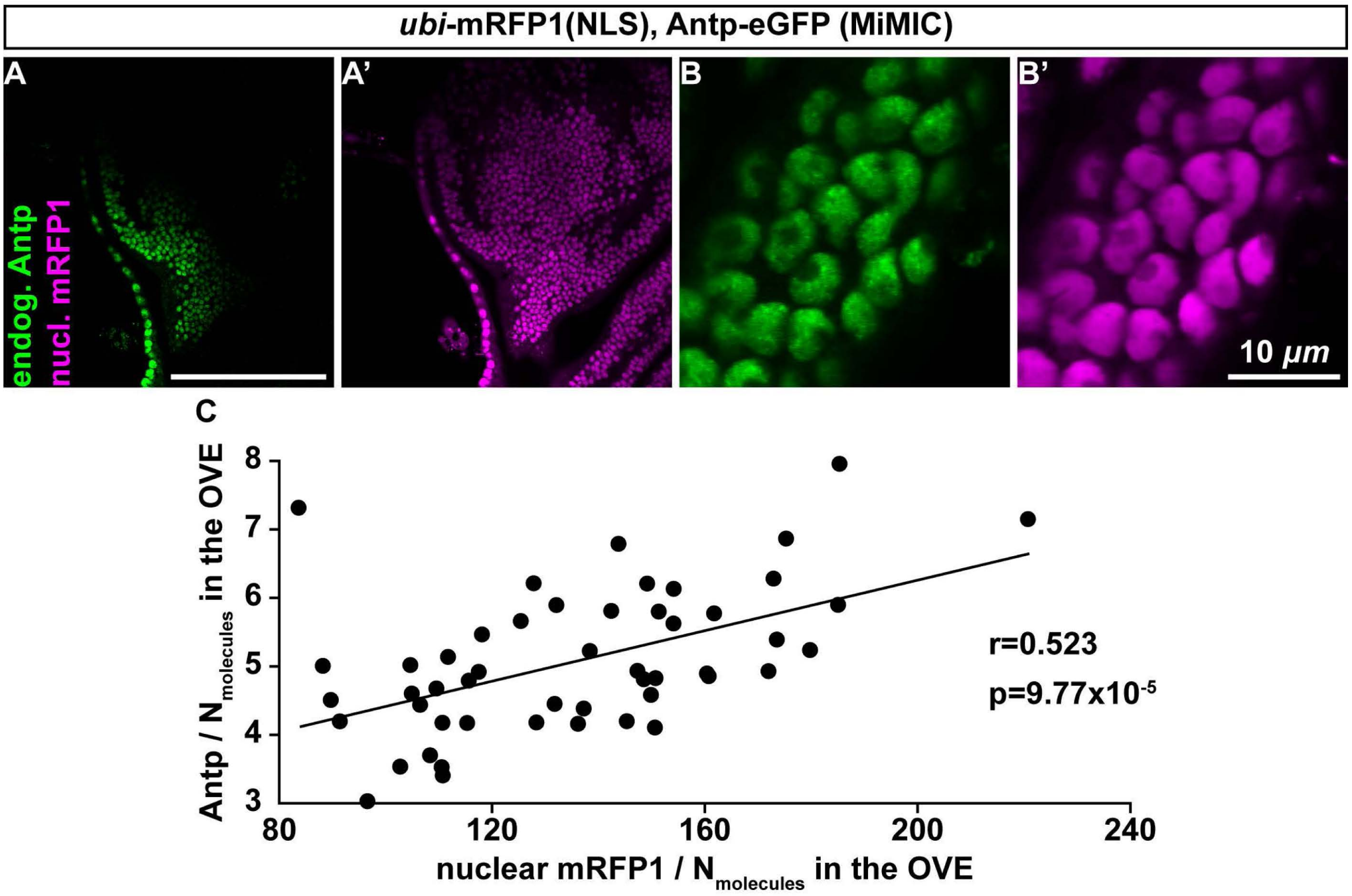

Figure S12. Investigation of extrinsic variability in the endogenous *Antp-eGFP* expression domain. (A-B') Live imaging of a wing disc notum, where nuclear mRFP1 protein is highly expressed from a constitute enhancer (*ubi*-mRFP1(NLS)), alongside with endogenous *Antp-eGFP*. FCS measurements were performed in the region of high co-expression of *Antp* and *mRFP1*. (B-B') Higher magnification of cells as in (A-A'). Note the uneven distribution of Antp in the nuclei and the formation of sites of accumulation in (B). (C) Plot of the concentration of Antp-eGFP (expressed as number of fluorescent molecules in the Observation Volume Element (OVE)). The correlation coefficient r was calculated to be $r = 0.523$ and the p-value to be *P* (correlation) $= 9.77 \times 10^{-5}$. Scale bars denote $100\ \mu m$, unless otherwise indicated.

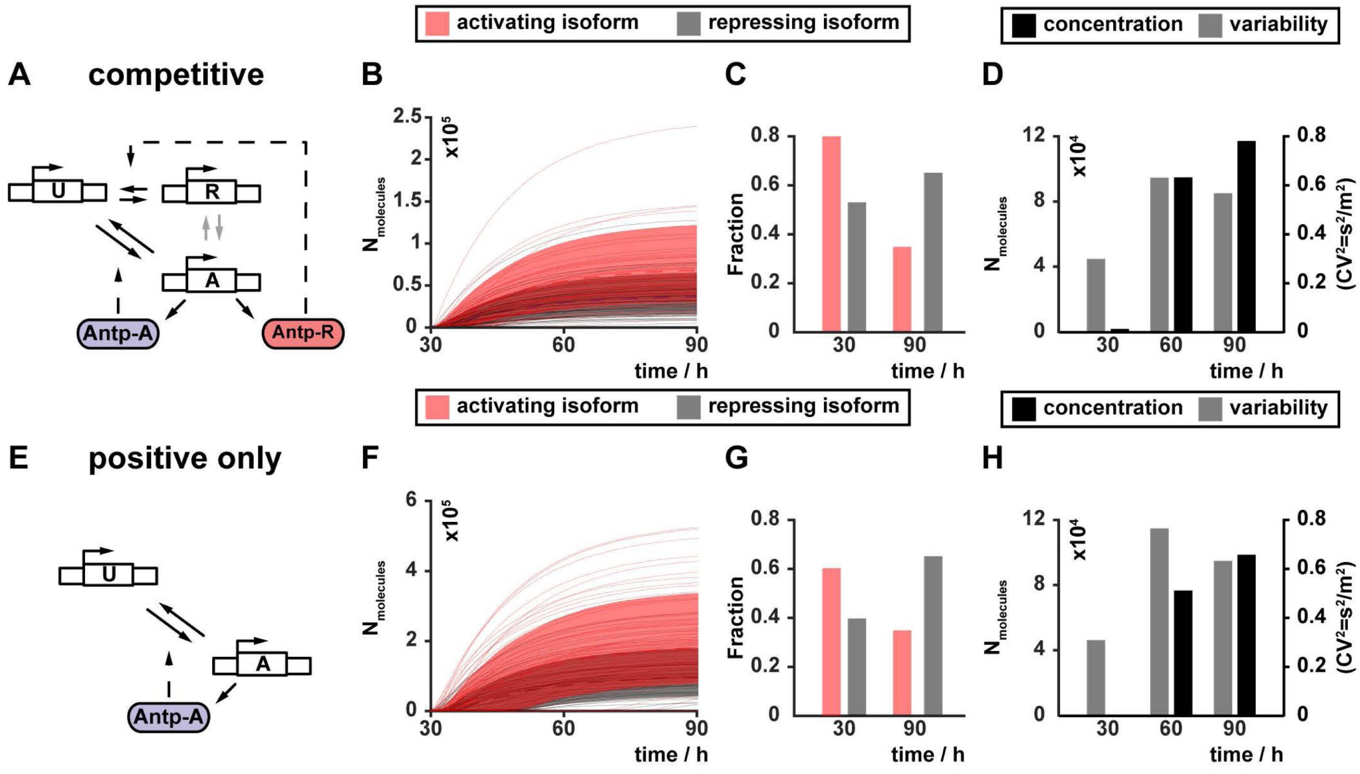

Figure S13. Models of *Antp* auto-regulation. (A-D) Competition of Antp binding, whereby state "A" can be reached only through the unbound state "U" in (A), results in increase in Antp protein numbers (D) without decrease in variability (grey bars in (D)). Trajectories of individual simulations are presented in (B) and the distribution of the Antp isoforms, predicted by the model, in (C). (E-H) Requirement of the negative feedback for suppression of variability. In the absence of the state "R" (E), concentration increases (H), but variability also increases rather than being suppressed (grey bars in (H)). Trajectories of individual simulations are presented in (F) and the distribution of the Antp isoforms, predicted by the model, in (G).

Development • Supplementary information

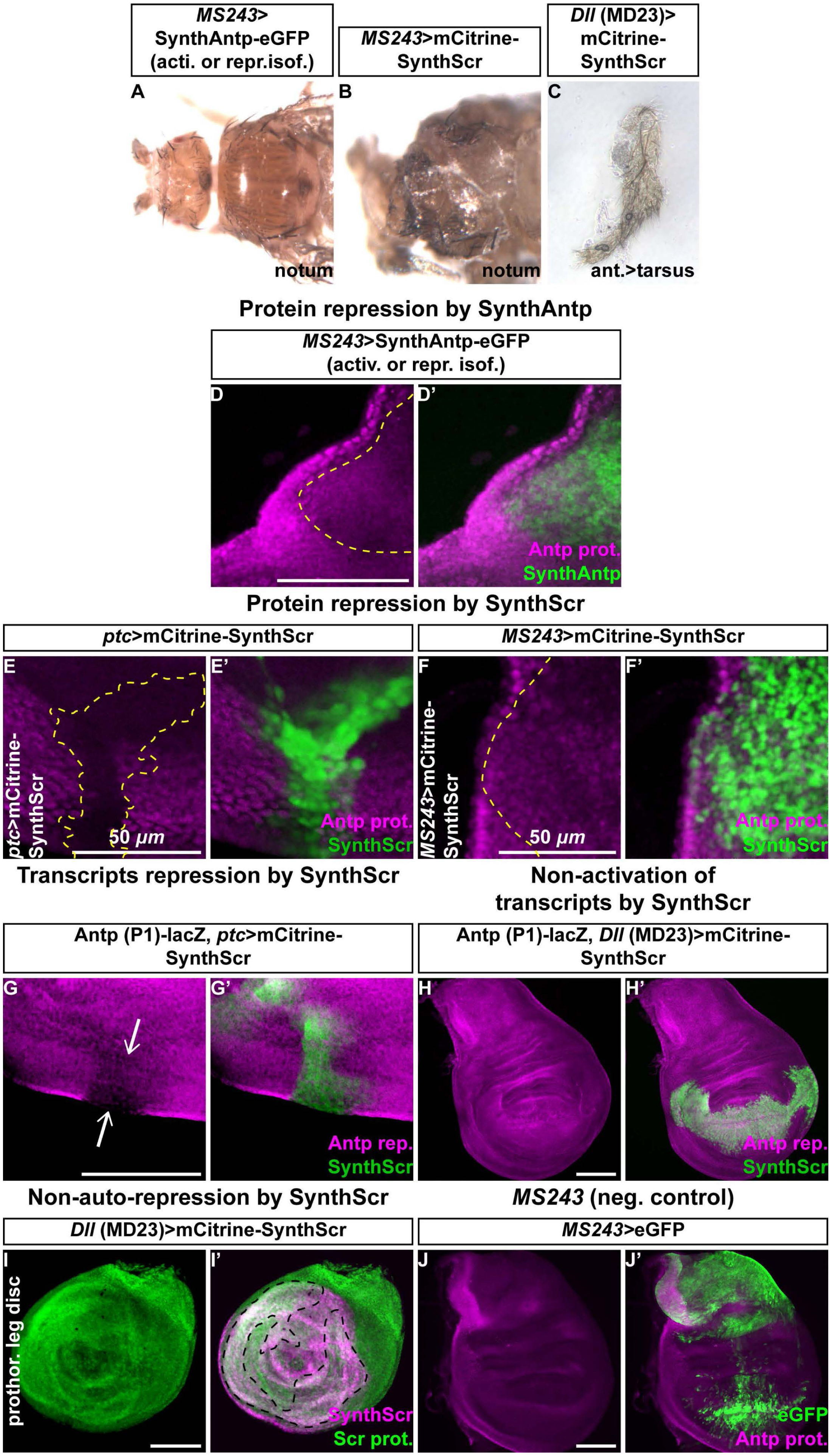
MS243>
SynthAntp-eGFP
(acti. or repr.isof.)
MS243>mCitrine-
SynthScr
Dll (MD23)>
mCitrine-
SynthScr
A
B
C
notum
notum
ant.>tarsus
Protein repression by SynthAntp
MS243>SynthAntp-eGFP
(activ. or repr. isof.)
D
D'
Antp prot.
SynthAntp
Protein repression by SynthScr
ptc>mCitrine-SynthScr
MS243>mCitrine-SynthScr
E
E'
F
F'
ptc>mCitrine-
SynthScr
50 μm
Antp prot.
SynthScr
MS243>mCitrine-
SynthScr
50 μm
Antp prot.
SynthScr
Transcripts repression by SynthScr
Non-activation of
transcripts by SynthScr
Antp (P1)-lacZ, ptc>mCitrine-
SynthScr
Antp (P1)-lacZ, Dll (MD23)>mCitrine-
SynthScr
G
G'
H
H'
Antp rep.
SynthScr
Antp rep.
SynthScr
Non-auto-repression by SynthScr
MS243 (neg. control)
Dll (MD23)>mCitrine-SynthScr
MS243>eGFP
I
I'
J
J'
prothor. leg disc
SynthScr
Scr prot.
eGFP
Antp prot.

Figure S14. Controls of *Antp* model predictions and Scr-mediated perturbations. (A-C) Perturbations of the model system in (Fig. 5A) by overexpression of Antp long or short linker isoforms or an exogenous Antp repressor (Scr). Overexpression of activating or repressing SynthAntp-eGFP isoforms by *MS243*-Gal4 results in normal development of the fly notum (A), whereas induction of an exogenous repressor (mCitrine-SynthScr) results in severe malformations, indicated by developmental defects of the adult cuticle (B). Flies of both genotypes in (A) and (B) die as pharate adults. (C) Induction of *mCitrine-SynthScr* in the antennal disc results in complete transformations of antenna to tarsus as the induction of *SynthAntp-eGFP* (Fig. 6A). (D-D') *MS243*-Gal4-mediated expression of repressing or activating SynthAntp isoforms results in repression of the Antp endogenous protein in the notum region of the wing disc. (E-E') Ectopic expression of SynthScr in the wing disc using *ptc*-Gal4 results in drastic reduction of endogenous Antp protein levels. (F-F') Ectopic expression of SynthScr by *MS243*-Gal4 in the notum results in repression of the Antp protein. (G-G') SynthScr represses *Antp* at the transcriptional level, as indicated by the absence of transcription of the *Antp* P1 reporter (white arrows in (G)). (H-H') Unlike SynthAntp, SynthScr is not able to activate the *Antp* P1 promoter reporter transcription (H), when induced by *Dll* (MD23)-Gal4. (I-I') SynthScr is not able to downregulate its own endogenous protein levels upon overexpression by *Dll* (MD23)-Gal4. Dashed line in (I') outlines the region of high overlap between the overexpressed SynthScr and endogenous Scr stainings. (J-J') Negative control staining for the induction of eGFP in the wing disc notum by *MS243*-Gal4, which fails to repress endogenous Antp protein. Dashed lines in (D), (E) and (F) outline the regions of ectopic overexpression of *SynthAntp* or *SynthScr*, where endogenous *Antp* is repressed, whereas the dashed line in (I') outlines the region of overlap between SynthScr overexpression and endogenous expression of the Scr protein, where no repression is observed. Scale bars denote $100\ \mu m$, unless otherwise indicated.

Development • Supplementary information

Supplementary Materials and Methods

Background on Fluorescence Microscopy Imaging and FCS

Two individually modified instruments (Zeiss, LSM 510 and 780, ConfoCor 3) with fully integrated FCS/CLSM optical pathways were used for imaging. The detection efficiency of CLSM imaging was significantly improved by the introduction of APD detectors. As compared to PMTs, which are normally used as detectors in conventional CLSM, the APDs are characterized by higher quantum yield and collection efficiency – about 70 % in APDs as compared to 15 – 25 % in PMTs, higher gain, negligible dark current and better efficiency in the red part of the spectrum. Enhanced fluorescence detection efficiency enabled image collection using fast scanning ($1-5\ \mu s/\mathrm{pixel}$). This enhances further the signal-to-noise-ratio by avoiding fluorescence loss due to triplet state formation, enabling fluorescence imaging with single-molecule sensitivity. In addition, low laser intensities ($150–750\ \mu W$) could be applied for imaging, significantly reducing the photo-toxicity (Vukojevic et al., 2008).

FCS measurements are performed by recording fluorescence intensity fluctuations in a very small, approximately ellipsoidal observation volume element (OVE) (about $0.2\ \mu m$ wide and $1\ \mu m$ long) that is generated in imaginal disc cells by focusing the laser light through the microscope objective and by collecting the fluorescence light through the same objective using a pinhole in front of the detector to block out-of-focus light. The fluorescence intensity fluctuations, caused by fluorescently labeled molecules passing through the OVE are analyzed using temporal autocorrelation analysis.

In temporal autocorrelation analysis we first derive the autocorrelation function $G(\tau)$:

$$G(\tau) = 1 + \frac{\langle \delta I(t) \cdot \delta I(t+\tau) \rangle}{\langle I(t) \rangle^2} \quad \text{(S1)},$$

where $\delta I(t) = I(t) - \langle I(t) \rangle$ is the deviation from the mean intensity at time t and $\delta I(t+\tau) = I(t+\tau) - \langle I(t) \rangle$ is the deviation from the mean intensity at time $t+\tau$. For further analysis, an autocorrelation curve is derived by plotting $G(\tau)$ as a function of the lag time, i.e. the autocorrelation time τ.

To derive information about molecular numbers and their corresponding diffusion time, the experimentally obtained autocorrelation curves are compared to autocorrelation functions derived for different model systems, and the model describing free three dimensional (3D) diffusion of two components and triplet formation was identified as the simplest and best suited for fitting the experimentally derived autocorrelation curves, and was used throughout:

$$G(\tau) = 1 + \frac{1}{N}\left(\frac{1-y}{\left(1+\frac{\tau}{\tau_{D_1}}\right)\cdot\sqrt{1+\frac{w_{xy}^2\tau}{w_z^2\tau_{D_1}}}} + \frac{y}{\left(1+\frac{\tau}{\tau_{D_2}}\right)\cdot\sqrt{1+\frac{w_{xy}^2\tau}{w_z^2\tau_{D_2}}}} \right)\cdot\left(1+\frac{T}{1-T}\cdot e^{-\frac{\tau}{\tau_T}}\right)$$

(S2)

In the above equation, *N* is the average number of molecules in the OVE; *y* is the fraction of the slowly moving Antp-eGFP molecules; τ_{D_1} is the diffusion time of the free Antp-eGFP molecules; τ_{D_2} is the diffusion time of Antp-eGFP molecules undergoing nonspecific interactions with the DNA; w_{xy} and w_z are radial and axial parameters, respectively, related to spatial properties of the OVE; T is the average equilibrium fraction of molecules in the triplet state; and τ_T the triplet correlation time

Development • Supplementary information

related to rate constants for intersystem crossing and the triplet decay. Spatial properties of the detection volume, represented by the square of the ratio of the axial and radial parameters ($\left(\frac{w_z}{w_{xy}}\right)^2$), are determined in calibration measurements performed using a solution of Rhodamine 6G for which the diffusion coefficient (D) is known to be $D_{Rh6G} = 4.1 \cdot 10^{-10}\, m^2 s^{-1}$ (Muller et al., 2008). The diffusion time, τ_D, measured by FCS, is related to the translation diffusion coefficient *D* by:

$$\tau_D = \frac{w_{xy}^2}{4D} \text{ (S3).}$$

To establish that Antp molecules diffusing through the OVE are the underlying cause of the recorded fluorescence intensity fluctuations, we plotted the characteristic decay times τ_{D1} and τ_{D2}, obtained by FCS, as a function of the total concentration of Antp molecules (Supplemental Fig. S2). We observed that both characteristic decay times remain stable for increasing total concentration of Antp molecules, signifying that the underlying process triggering the fluorescence intensity fluctuations is diffusion of fluorescent Antp molecules through the OVE (which is independent of the total concentration of Antp molecules).

In order to ascertain that the interpretation and fitting of FCS curves is correct, we have: (1) tested several laser intensities in our FCS measurements and have utilized the highest laser intensity, for which the highest counts per second and molecule (CPSM) were obtained, while photobleaching was not observed; (2) we have established that CPSM do not change among FCS measurements performed in cells expressing *Antp* endogenously, or overexpressed with different Gal4 drivers. Moreover, we have previously shown that both characteristic decay times increase when the size of the OVE is increased (Fig. 4 in (Vukojevic et al., 2010)). Together, these lines of evidence indicate that both short and long characteristic decay times are generated by molecular diffusion rather than by photophysical and/or chemical processes such as eGFP protonation/deprotonation; (3) we have ascertained that the long characteristic decay time of our FCS measurements is not the result of photobleaching and that differences in the relative amplitudes of the fast and slow diffusing components reflect differences in their concentrations among cells.

While we have taken all possible precautions to ascertain that the correct model for FCS data fitting is applied, some inevitable limitations still remain. For example, FCS cannot account for Antp molecules with irreversibly photobleached fluorophores or with fluorophores residing for various reasons in dark states. In addition, FCS cannot account for Antp molecules associated with large immobile structures, such as specifically bound Antp molecules. These molecules contribute to the overall background signal, but they do not give rise to fluorescence intensity fluctuations. As a consequence, transcription factor concentration can be somewhat underestimated by FCS. In contrast, the number of transcription factor molecules may also be overestimated by FCS, when high background signal as compared to fluorescence intensity may lead to an artificially low amplitude of FCS curves, and, hence, overestimation of molecular numbers. To avoid artifacts due to photobleaching, the incident laser intensity was kept as low as possible but sufficiently high to allow high signal-to-noise ratio. This is because photobleaching of fluorophores may induce errors in the measurements of molecular numbers and lateral diffusion, yielding both smaller number of molecules and shorter values of τ_D, and hence apparently larger diffusion coefficients. Finally, contribution of brightness, *i.e.* brightness squared, to the correlation function was not analyzed, which may in turn affect quantification of Antp numbers.

Development • Supplementary information

Calculation of the concentration of endogenous TFs and average number of molecules in imaginal disc cell nuclei from FCS measurements (exemplified for Antp)

Experimentally derived FCS curves were analyzed by fitting, using the model function for free three-dimensional diffusion of two components with triplet formation, equation (S2), to derive the average number of molecules in the OVE (N); the diffusion time of the free Antp-eGFP molecules (τ_{D_1}); the diffusion time of Antp-eGFP molecules undergoing interactions with the DNA (τ_{D_2}); and the relative fraction of Antp-eGFP molecules that are engaged in interactions with chromatin and therefore move slowly (y).

In order to translate the average number of molecules in the OVE (N) into molar concentration, the size of the OVE, *i.e.* the axial and radial parameters (w_z and w_{xy}, respectively) were determined in calibration experiments with Alexa488 or Rhodamine 6G dyes, using equation (S3). The volume of the OVE, approximated by a prolate ellipsoid, was determined as follows:

$$V_{OVE} = \pi^{\frac{3}{2}} \cdot w_{xy}^2 \cdot z_0 = 5.57 \cdot 0.1847^{\mathbf{2}} \cdot 1 = 0.223 \cdot 10^{-18}\, m^3 = 0.22 \cdot 10^{-15} L \text{ (S4).}$$

Thereafter, the average number of molecules in the OVE (N) was converted into molar concentration (C) using the relationship:

$$C = \frac{N}{N_A \cdot V_{OVE}} \text{ (S5),}$$

where N_A is the Avogadro number ($6.022 \cdot 10^{23} mol^{-1}$), which indicated that one molecule in the OVE corresponds on the average to $8.74\, nM$ concentration of fluorescent molecules in the nucleus.

Finally, the concentration of non-specifically bound TF molecules ($[DNA - Antp - eGFP]_{ns}$) was calculated by multiplying the relative amplitude of the second component (y), determined by fitting the experimental autocorrelation curves with the model function (S2), with the total concentration of Antp-eGFP, $C = [Antp - eGFP]_0$, which was determined from the amplitude of the autocorrelation curve at zero lag time:

$$[DNA - Antp - eGFP]_{ns} = y \cdot [DNA - Antp - eGFP]_0 \text{ (S6).}$$

The concentration of non-specifically bound TF molecules ($[DNA - Antp - eGFP]_{ns}$) was then plotted as a function of the total concentration of Antp-eGFP ($[Antp - eGFP]_0$) to yield the graphs shown in Supplemental Fig. S10C,D.

In order to estimate the total number of molecules in the wing disc imaginal cell nuclei, we applied the following calculation. The wing disc nuclei within the *Antp* expression domain (prescutum precursors) are not spherical, but rather ellipsoidal. Their axes were determined by fluorescence imaging to be $1.4\, \mu m$ in the transverse dimension and $2.8\, \mu m$ in the longitudinal. The volume of the nucleus was approximated by the volume of a prolate ellipsoid:

$$V_{nucleus} = \frac{4}{3}\pi a^2 b = \frac{4}{3} \cdot 3.14 \cdot (1.4 \cdot 10^{-6})^2 \cdot 2.8 \cdot 10^{-\mathbf{6}}\, m^3 = 22.99 \cdot 10^{-18}\, m^3 = 22.99 \cdot 10^{-15}\, L \text{ (S7).}$$

Therefore, the OVE represents roughly $1/121$ of the nuclear volume:

$$\frac{V_{nucleus}}{V_{OVE}} = 121 \text{ (S8)}$$

and the number of molecules in Antp-eGFP nuclei is on the average $57.37 \cdot 121 \approx 6942$ molecules in third instar wing and $127 \cdot 121 \approx 15367$ in third instar leg discs.

Generalizing, with known axial and radial parameters of the OVE and calculation of the transverse and longitudinal dimensions of the nucleus, the total number of molecules of transcription factor in the nucleus can be estimated:

Development • Supplementary information

$$N_{TF\ molecules}^{total} = \frac{V_{nucleus}}{V_{OVE}} \cdot N_{TF\ molecules}^{OVE} = \frac{\frac{4}{3}\pi a^2 b N_{TF\ molecules}^{OVE}}{\pi^{\frac{3}{2}} \cdot w_{xy}^2 \cdot z_0} = \frac{4a^2 b N_{TF\ molecules}^{OVE}}{3\sqrt{\pi} \cdot w_{xy}^2 \cdot z_0} \text{ (S9)}$$

Calculation of the ratio of apparent Antp dissociation constant for short and long linker Antp isoforms from FCS measurements on ectopically expressed Antp

Antp undergoes both specific and non-specific interactions with DNA, with non-specific interactions preceding the specific ones and effectively assisting the binding to a specific target site by facilitated diffusion (Halford and Marko, 2004). The searching for specific binding sites can be described as a two-step process of consecutive reactions (Vukojevic et al., 2010):

$$DNA_{ns} + Antp - eGFP \underset{k_{-ns}}{\overset{k_{ns}}{\Leftrightarrow}} (DNA - Antp - eGFP)_{ns}$$

$$(DNA - Antp - eGFP)_{ns} + DNA_s \underset{k_{-s}}{\overset{k_s}{\Leftrightarrow}} (DNA - Antp - eGFP)_s \text{ (S10).}$$

The turnover rate for the non-specific complex is:

$$\frac{d[(DNA-Antp-eGFP)_{ns}]}{dt} = k_{ns} \cdot [DNA_{ns}] \cdot [Antp - eGFP] - (k_{-ns} + k_s \cdot [DNA_s]) \cdot [(DNA - Antp - eGFP)_{ns}] + k_{-s} \cdot [(DNA - Antp - eGFP)_s] \text{ (S11).}$$

Assuming a quasi-steady state approximation:

$$\frac{d[(DNA-Antp-eGFP)_{ns}]}{dt} = 0 \text{ (S12),}$$

$$(k_{-ns} + k_s \cdot [DNA_s]) \cdot [(DNA - Antp - eGFP)_{ns}] = k_{ns} \cdot [DNA_{ns}] \cdot [Antp - eGFP] + k_{-s} \cdot [(DNA - Antp - eGFP)_s] \text{ (S13).}$$

Using the mass balance equation to express the concentration of the free TF:

$$[Antp - eGFP] = [Antp - eGFP]_0 - [(DNA - Antp - eGFP)_{ns}] - [(DNA - Antp - eGFP)_s] \text{ (S14)}$$

and assuming that:

$$[DNA]_{ns} \approx [DNA]_0 \text{ (S15),}$$

equation (S13) becomes:

$$(k_{-ns} + k_s \cdot [DNA_s]) \cdot [(DNA - Antp - eGFP)_{ns}] = k_{ns} \cdot [DNA]_0 \cdot ([Antp - eGFP]_0 - [(DNA - Antp - eGFP)_{ns} - [(DNA - Antp - eGFP)_s]) + k_{-s} \cdot [(DNA - Antp - eGFP)_s] \text{ (S16),}$$

$$(k_{-ns} + k_s \cdot [DNA_s] + k_{ns} \cdot [DNA]_0) \cdot [(DNA - Antp - eGFP)_{ns}] = k_{ns} \cdot [DNA]_0 \cdot ([Antp - eGFP]_0 - [(DNA - Antp - eGFP)_s]) + k_{-s} \cdot [(DNA - Antp - eGFP)_s] \text{ (S17),}$$

$$(k_{-ns} + k_s \cdot [DNA_s] + k_{ns} \cdot [DNA]_0) \cdot [(DNA - Antp - eGFP)_{ns}] = k_{ns} \cdot [DNA]_0 \cdot [Antp - eGFP]_0 - (k_{ns} \cdot [DNA]_0 - k_{-s}) \cdot [(DNA - Antp - eGFP)_s] \text{ (S18),}$$

$$[(DNA - Antp - eGFP)_{ns}] = \frac{k_{ns} \cdot [DNA]_0}{k_{-ns} + k_s \cdot [DNA_s] + k_{ns} \cdot [DNA]_0} \cdot [Antp - eGFP]_0 - \frac{k_{ns} \cdot [DNA]_0 - k_{-s}}{k_{-ns} + k_s \cdot [DNA_s] + k_{ns} \cdot [DNA]_0} \cdot [(DNA - Antp - eGFP)_s] \text{ (S19).}$$

According to equation (S19) and the FCS data presented in Supplemental Fig. S10, the slope of the linear dependence for:

a) the short linker Antp isoform gives:

$$\frac{k_{ns}^{short\ linker} \cdot [DNA]_0}{k_{-ns}^{short\ linker} + k_s^{short\ linker} \cdot [DNA_s] + k_{ns}^{short\ linker} \cdot [DNA]_0} = 0.34 \text{ (S20)}$$

and the intercept:

Development • Supplementary information

$$\frac{k_{ns}^{short\ linker}\cdot[DNA]_0-k_{-s}^{short\ linker}}{k_{-ns}^{short\ linker}+k_{s}^{short\ linker}\cdot[DNA_s]+k_{ns}^{short\ linker}\cdot[DNA]_0}\cdot[(DNA-Antp-eGFP)_s]=5.31\ nM$$ (S21).

If $k_{-s}^{short\ linker}$ is small compared to $k_{ns}^{short\ linker}\cdot[DNA]_0$ and can therefore be neglected, then:

$$0.34\cdot[(DNA-Antp-eGFP)_s]=5.31\ nM$$ (S22).

Thus, the concentration of specific complex between Antp-eGFP and DNA in the wing disc cell nuclei can be estimated to be:

$$[(DNA-Antp-eGFP)_s]=15.62\ nM$$ (S23).

The average concentration of free-diffusing Antp-eGFP molecules is determined as follows:

$$[Antp-eGFP]_{free}=[Antp-eGFP]_0-[(DNA-Antp-eGFP)_{ns}]-[(DNA-Antp-eGFP)_s]=[Antp-eGFP]_0-(0.34\cdot[Antp-eGFP]_0-5.31)-[(DNA-Antp-eGFP)_s]=785.28-0.34\cdot785.28+5.31-15.62=507.97nM$$ (S24).

Using the experimentally determined concentration of specific DNA–Antp-eGFP complexes (equation (S23)), we could estimate the dissociation constant for the specific DNA–Antp-eGFP, as a function of the total concentration of specific Antp binding sites, to be:

$$K_{d,Antp}^{short\ linker}=\frac{[DNA_s]_{free}\cdot[Antp-eGFP]_{free}}{[(DNA-Antp-eGFP)_s]}=\frac{[DNA_s]_{free}\cdot507.97}{15.62}\approx([DNA_s]_{free}\cdot32.52)\ nM$$ (S25).

b) The long linker Antp isoform gives:

$$\frac{k_{ns}^{long\ linker}\cdot[DNA]_0}{k_{-ns}^{long\ linker}+k_{s}^{long\ linker}\cdot[DNA_s]+k_{ns}^{long\ linker}\cdot[DNA]_0}=0.24$$ (S26).

and the intercept:

$$\frac{k_{ns}^{long\ linker}\cdot[DNA]_0-k_{-s}^{long\ linker}}{k_{-ns}^{long\ linker}+k_{s}^{long\ linker}\cdot[DNA_s]+k_{ns}^{long\ linker}\cdot[DNA]_0}\cdot[(DNA-Antp-eGFP)_s]=3.28\ nM$$ (S27).

If $k_{-s}^{long\ linker}$ is small compared to $k_{ns}^{long\ linker}\cdot[DNA]_0$ and can therefore be neglected, then:

$$0.24\cdot[(DNA-Antp-eGFP)_s]=3.28\ nM$$ (S28).

Thus, the concentration of specific complex between Antp-eGFP and DNA in the wing disc cell nuclei can be estimated to be:

$$[(DNA-Antp-eGFP)_s]=13.67\ nM$$ (S29).

The average concentration of free-diffusing Antp-eGFP molecules is determined as follows:

$$[Antp-eGFP]_{free}=[Antp-eGFP]_0-[(DNA-Antp-eGFP)_{ns}]-[(DNA-Antp-eGFP)_s]=[Antp-eGFP]_0-(0.24\cdot[Antp-eGFP]_0-3.28)-[(DNA-Antp-eGFP)_s]=1382.95-0.24\cdot785.28+3.28-13.67=1040.65nM$$ (S30).

Using the experimentally determined concentration of specific DNA–Antp-eGFP complexes (equation (S29)), we could estimate the dissociation constant for the specific DNA–Antp-eGFP, as a function of the total concentration of specific Antp binding sites, to be:

$$K_{d,Antp}^{long\ linker}=\frac{[DNA_s]_{free}\cdot[Antp-eGFP]_{free}}{[(DNA-Antp-eGFP)_s]}=\frac{[DNA_s]_{free}\cdot1040.65}{13.67}\approx([DNA_s]_{free}\cdot76.13)\ nM$$ (S31).

From equations (S25) and (S31), we could calculate the ratio of the apparent equilibrium dissociation constants for specific interactions to be:

Development • Supplementary information

$$\frac{K_{d,Antp}^{long\ linker}}{K_{d,Antp}^{short\ linker}} = \frac{[DNA_s]_{free}^{long\ linker} \cdot 76.13}{[DNA_s]_{free}^{short\ linker} \cdot 32.52} = 2.34 \cdot \frac{([DNA]_0 - [(DNA-Antp-eGFP)_s]_{long\ linker})}{([DNA]_0 - [(DNA-Antp-eGFP)_s]_{short\ linker})} =$$

$$2.34 \frac{[DNA]_0 - 13.67}{[DNA]_0 - 15.62} \text{ (S32).}$$

Therefore:

$$K_{d,Antp}^{s,long\ linker} > 2.34 \cdot K_{d,Antp}^{s,short\ linker} \text{ (S33),}$$

independently of the total concentration of Antp binding sites in the nucleus. Although the affinities of the ratio of the equilibrium dissociation constants does not depend on the total concentration of binding sites for Antp ($[DNA]_0$), its value is required for the calculation. For values close to $15.62\,nM$ ($[DNA]_0 \to 15.62\,nM$) with $[DNA]_0 > 15.62\,nM$, the ratio of the apparent equilibrium dissociation constants will be high:

$$\lim_{[DNA]_0 \to 15.62} \left(2.34 \frac{[DNA]_0 - 13.67}{[DNA]_0 - 15.62}\right) = +\infty \text{ (S34),}$$

indicating that, in this case, the short linker isoform will bind the Antp binding sites with much higher affinity than the long linker isoform.

In contrast, for considerably higher values of $[DNA]_0$ than $15.62\,nM$ ($[DNA]_0 \to +\infty$), the ratio of apparent equilibrium dissociation constants will be:

$$\lim_{[DNA]_0 \to +\infty} \left(2.34 \frac{[DNA]_0 - 13.67}{[DNA]_0 - 15.62}\right) = \lim_{[DNA]_0 \to +\infty} \left(2.34 \frac{[DNA]_0\left(1 - \frac{13.67}{[DNA]_0}\right)}{[DNA]_0\left(1 - \frac{15.62}{[DNA]_0}\right)}\right) =$$

$$\lim_{[DNA]_0 \to +\infty} \left(2.34 \frac{1 - \frac{13.67}{[DNA]_0}}{1 - \frac{15.62}{[DNA]_0}}\right) = 2.34 \text{ (S35),}$$

indicating a roughly 2.5fold higher affinity of the short linker repressive isoform.

In addition, equations (S20) and (S26) contain information about the ratio of the apparent equilibrium dissociation constants for nonspecific interactions [(Vukojevic et al., 2010), Fig. 5, solid red line *versus* dashed red line]. Thus, the slopes of the linear regression lines (Supplemental Fig. S10C-D'), give:

$$\frac{k_{ns}^{short\ linker} \cdot [DNA]_0}{k_{-ns}^{short\ linker} + k_{ns}^{short\ linker} \cdot [DNA]_0} = 0.34 \text{ (S36)}$$

$$\frac{k_{ns}^{long\ linker} \cdot [DNA]_0}{k_{-ns}^{long\ linker} + k_{ns}^{long\ linker} \cdot [DNA]_0} = 0.24 \text{ (S37)}$$

From these relationships, the ratio of the apparent equilibrium dissociation constants for nonspecific interactions can be estimated to be:

$$K_{d,Antp}^{ns,long\ linker} > 1.63 \cdot K_{d,Antp}^{ns,short\ linker} \text{ (S38).}$$

Thus, our analysis shows that the short linker, which is the preferentially repressing isoform, binds with higher affinity (lower K_d) to both specific and nonspecific binding sites on the DNA [(S33) and (S38), respectively]. This, in turn, implies that the short linker is also more efficient in searching for specific TF binding sites, as evident from the lower dissociation constant for nonspecific DNA interactions of the short linker isoform (Sela and Lukatsky, 2011; Soltani et al., 2015), and that it binds with lower apparent dissociation constant to specific binding sites on the DNA.

Stochastic modeling of Antennapedia expression

In the following, we develop a simple mathematical model that is able to explain the behavior of *Antp* expression at early and late developmental stages. The *Antp* promoter is modeled as a continuous-time Markov chain with three distinct transcriptional states. In the absence of Antp, the promoter is in an unbound state ("U"), in which transcription is inactive. From this state, the promoter can switch to a transcriptionally active state "A" at a rate, which we consider to be proportional to the concentration of the long-linker, activating isoform of Antp. Analogously, repression of

Development • Supplementary information

the promoter by the short-linker isoform of Antp is modeled by an additional transcriptionally inactive state "R", which can be reached from state "U" at a rate proportional to the concentration of that isoform. The corresponding reverse transitions from states "R" and "A" back into state "U" are assumed to happen at a constant rate k. Since the activating isoform can potentially also repress the promoter, we assume that state "R" can be reached also from the active state "A". Similarly, we model a potential link also in the reverse direction from state "A" to "R". Depending on the model variant, we consider this transition to happen either at a constant rate k (competitive promoter model) or at a rate proportional to the concentration of the repressing isoform of Antp (non-competitive promoter model). In the latter case, repression through short-linker isoforms can take place even if a long-linker isoform is already bound to the promoter. As we have demonstrated in Fig. 5A,B, the two model variants yield qualitative differences in *Antp* expression. For the sake of illustration, the following description focuses on the non-competitive model variant but we remark that the competitive model can be derived analogously.

At a particular time point t, the transcription rate of *Antp* is determined by the current state of the promoter, i.e., $\lambda(t)\epsilon\{0,\ \lambda_A,\ 0\}$, with λ_A as the transcription rate associated with state "A". In line with our experimental findings, we assume that transcripts are spliced into the activating and repressing isoforms at different rates ρ_A and ρ_R, respectively. This allows us to capture the imbalance between the two isoforms that was revealed by our FCS data. The overall expression rates for the two isoforms of Antp are then given by $h_A(t) = \lambda(t)Z\rho_A$ and $h_R(t) = \lambda(t)Z\rho_R$, whereas Z is a random variable that accounts for extrinsic variability in gene expression rates (Zechner et al., 2012). In all of our analyses, we model Z as a Gamma-distributed random variable $Z\sim\Gamma(\alpha, \beta)$ with α and β as shape and inverse scale parameters of that distribution. In summary, we describe the auto-regulatory circuit of *Antp* expression by a Markovian reaction network of the form:

$$U \underset{k}{\overset{\gamma_A X_A(t)^n}{\rightleftharpoons}} A$$

$$U \underset{k}{\overset{\gamma_R X_R(t)^n}{\rightleftharpoons}} R$$

$$A \underset{k}{\overset{\gamma_R X_R(t)^n}{\rightleftharpoons}} R$$

$$\emptyset \xrightarrow{h_A(t)} X_A \xrightarrow{1/\tau} \emptyset$$

$$\emptyset \xrightarrow{h_R(t)} X_R \xrightarrow{1/\tau} \emptyset$$

(S39),

with $X_A(t)$and $X_R(t)$ as the concentration of the activating and repressing isoforms of Antp, τ as the protein half-live and n as a coefficient accounting for cooperativity in the binding of Antp to the promoter. The initial conditions $X_A(0)$ and $X_R(0)$ were drawn randomly in accordance with our concentration measurements at early stages. In particular, we assume that the total amount of Antp X_{tot} in each cell is drawn from a negative binomial distribution such that $X_{tot}\sim\mathcal{NB}(r,p)$, with $\mu_X = r(1-p)/p$ and $\eta_x^2 = 1/r(1-p)$ as the mean and squared coefficient of variation of this distribution. The total number of Antp molecules was then randomly partitioned into fractions of repressing and activating isoforms according to a Beta distribution. More specifically, we set $X_A(0) = WX_{tot}$ and $X_R(0) = (1-W)X_{tot}$ with $W\sim Beta(a,b)$. The parameters r, p, a and b were chosen based on our experimental data (see Table 1).

Due to the fact that *Antp* expression takes place at the timescale of several hours to days, we can further simplify our model from (S39). In particular, we can make

Development • Supplementary information

use of a quasi-steady state assumption (Rao and Arkin, 2003), by assuming that promoter switching due to binding and unbinding of the different Antp isoforms occurs at a much faster timescale than production and degradation of Antp. As a consequence, we can replace the stochastic gene expression rates of the two isoforms by their expected value, whereas the expectation is taken with respect to the quasi-stationary distribution of the three-state promoter model. More precisely, we have:

$$h_A\,(t) \approx \mathbb{E}[\lambda(t)]Z\rho_A$$
$$h_R\,(t) \approx \mathbb{E}[\lambda(t)]Z\rho_R$$
(S40),

with $\mathbb{E}[\lambda(t)] = P_U 0 + P_A\lambda_A + P_R 0 = P_A\lambda_A$ as the quasi-stationary probabilities of finding the promoter in state "U", "A" and "R", respectively. These probabilities can be derived from the generator matrix of the three-state promoter model, which reads:

$$Q = \begin{pmatrix} -\gamma_A X_A(t)^n - \gamma_R X_R(t)^n & k & k \\ \gamma_A X_A(t)^n & -k - \gamma_R X_R(t)^n & k \\ \gamma_R X_R(t)^n & \gamma_R X_R(t)^n & -2k \end{pmatrix} \text{(S41).}$$

Assuming that $X_A(t)$ and $X_R(t)$ remain roughly constant on the timescale of the promoter, the quasi-stationary distribution can be determined by the null-space of Q, which is given by:

$$P_{QSS} = \begin{pmatrix} P_U \\ P_A \\ P_R \end{pmatrix} = \begin{pmatrix} \frac{k}{(k+\gamma_A X_A(t)^n + \gamma_R X_R(t)^n)} \\ \frac{k(2\gamma_A X_A(t)^n + \gamma_R X_R(t)^n)}{(2k+\gamma_R X_R(t)^n)(k+\gamma_A X_A(t)^n + \gamma_R X_R(t)^n)} \\ \frac{\gamma_R X_R(t)^n}{2k+\gamma_R X_R(t)^n} \end{pmatrix} \text{(S42).}$$

Correspondingly, the expectation of $\lambda(t)$ becomes:

$$\mathbb{E}[\lambda(t)] = (0 \quad \lambda_A \quad 0)\begin{pmatrix} P_U \\ P_A \\ P_R \end{pmatrix} = \frac{k(2\gamma_A X_A(t)^n + \gamma_R X_R(t)^n)}{(2k + \gamma_R X_R(t)^n)(k + \gamma_A X_A(t)^n + \gamma_R X_R(t)^n)}$$
$$:= \bar{\lambda}(X_A(t), X_R(t)) \text{ (S43).}$$

The simplified model of *Antp* expression can then be compactly written as two coupled birth-and-death processes:

$$\emptyset \xrightarrow{\bar{h}_A(X_A(t),X_R(t))} X_A \xrightarrow{1/\tau} \emptyset$$
$$\emptyset \xrightarrow{\bar{h}_R(X_A(t),X_R(t))} X_R \xrightarrow{1/\tau} \emptyset$$
(S44),

with $\bar{h}_A(X_A(t), X_R(t)) = \bar{\lambda}(X_A(t), X_R(t))Z\rho_A$ and $\bar{h}_R(X_A(t), X_R(t)) = \bar{\lambda}(X_A(t), X_R(t))Z\rho_R$.

In all our simulation studies, the circuit from (S44) was simulated using the τ-leaping algorithm (Gillespie, 2007). In case of the perturbation experiments, small modifications to the model were made. Overexpression of either of the two isoforms was reflected by changing the initial conditions of Antp. In particular, we added to the overexpressed isoform a random number of molecules drawn from a negative binomial distribution with mean μ_O and squared coefficient of variation η_O^2 (see Table 1). To account for overexpression of an external repressor S, we introduced a fourth state in the promoter model, from which no expression can take place. This state is assumed to be reachable from any of the other three states at a rate $\gamma_S S(t)^{n_S}$ with $S(t)$ as the concentration of the external repressor at time t and n_S as a coefficient accounting for cooperativity in the binding of the repressor to the promoter. For simplicity, we assumed $n_S = n$ in our case studies. To account for cell-to-cell variability in the repressor concentration, the latter was initialized randomly according to a Poisson distribution, i.e., $S(t_0) \sim Pois(Z\mu_S)$ with μ_S as the average repressor abundance and Z

Development • Supplementary information

as the Gamma-distributed random variable defined above. Furthermore, repressor molecules were assumed to have an average lifetime of τ_S, i.e., $S \xrightarrow{\tau_S^{-1}} \emptyset$.

The corresponding reaction rates of *Antp* expression were determined analogously to equations (S41-S43). Table 1 summarizes the parameters used for each of the simulation studies.

Fly genotypes corresponding to fluorescence images in the supplemental Figures

Supplemental Fig. S1A: FlyFos018487(pRedFlp-Hgr)(ato37785::2XTY1-SGFP-V5-preTEV-BLRP-3XFLAG)dFRT
Supplemental Fig. S1B: FlyFos024884(pRedFlp-Hgr)(brk25146::2XTY1-SGFP-V5-preTEV-BLRP-3XFLAG)dFRT
Supplemental Fig. S1C: FlyFos030836(pRedFlp-Hgr)(salm30926::2XTY1-SGFP-V5-preTEV-BLRP-3XFLAG)dFRT
Supplemental Fig. S1: FlyFos029681(pRedFlp-Hgr)(yki19975::2XTY1-SGFP-V5-preTEV-BLRP-3XFLAG)dFRT
Supplemental Fig. S1E: w^{1118}; PBac(fkh-GFP.FPTB)VK00037/SM5
Supplemental Fig. S1F: *sd*-eGFP (FlyTrap, homozygous)
Supplemental Fig. S1G: w^{1118}; PBac(grh-GFP.FPTB)VK00033
Supplemental Fig. S1H: FlyFos018974(pRedFlp-Hgr)(Scr19370::2XTY1-SGFP-V5-preTEV-BLRP-3XFLAG)dFRT
Supplemental Fig. S1I: FlyFos015942(pRedFlp-Hgr)(sens31022::2XTY1-SGFP-V5-preTEV-BLRP-3XFLAG)dFRT
Supplemental Fig. S1J,K: Antp-eGFP (MiMIC) homozygous (line MI02272, converted to an artificial exon)
Supplemental Fig. S1L: w^{1118}; PBac(Abd-B-EGFP.S)VK00037/SM5
Supplemental Fig. S1M: w^{1118}; PBac(ey-GFP.FPTB)VK00033
Supplemental Fig. S1N: w^{1118}; PBac(ss-GFP.A.FPTB)VK00037
Supplemental Fig. S1O,P: w^{1118}; PBac(grn-GFP.FPTB)VK00037
Supplemental Fig. S3A,A': *Antp* P1-*lacZ*/TM3
Supplemental Fig. S3B,B': *Antp* P2-*lacZ*/CyO
Supplemental Fig. S3C,C': wild type
Supplemental Fig. S3D,D': *hs*-flp; *act5C*-FRT-yellow-FRT-Gal4, UAS-eGFP
Supplemental Fig. S3E,E': *hs*-flp/+; *act5C*-FRT-yellow-FRT-Gal4, UAS-eGFP/+; *Antp* P1-*lacZ*/+
Supplemental Fig. S3F,F': *hs*-flp/+; *act5C*-FRT-yellow-FRT-Gal4, UAS-eGFP/+; UAS-Antp long linker (full-length, untagged)/*Antp* P1-*lacZ*
Supplemental Fig. S3G,G': *Dll*-Gal4 (MD23)/+; UAS-Antp long linker (full-length, untagged), UAS-mRFP1(NLS)/ *Antp* P1-*lacZ*
Supplemental Fig. S3H,H': *Dll*-Gal4 (MD23)/+; UAS-mRFP1(NLS)/ *Antp* P1-*lacZ*
Supplemental Fig. S4A: *Dll*-Gal4 (MD23)/+; UAS-SynthAntp long linker-eGFP/+
Supplemental Fig. S4B: *ptc*-Gal4/+; UAS-SynthAntp long linker-eGFP/+
Supplemental Fig. S4C: *Dll*-Gal4 (MD713)/+; UAS-SynthAntp long linker-eGFP/+
Supplemental Fig. S4D,G,H,K: *69B*-Gal4/UAS-SynthAntp long linker-eGFP
Supplemental Fig. S4I,J,L: *69B*-Gal4/UAS- eGFP
Supplemental Fig. S6B: *Antp* P1-*lacZ/*TM6B
Supplemental Fig. S7A,A': *hs*-flp/+; *ubi*-FRT-mChery-FRT-Gal4/+; Antp-eGFP (MiMIC)/UAS-Antp long linker (full-length, untagged)
Supplemental Fig. S7B-C': *hs*-flp/+; *ubi*-FRT-mChery-FRT-Gal4/+; Antp-eGFP (MiMIC)/+

Development • Supplementary information

Supplemental Fig. S7D,D’: *hs*-flp/+; *act5C*-FRT-yellow-FRT-Gal4, UAS-eGFP/+; *Antp* P1-*lacZ*/UAS-Antp long linker (full-length, untagged)
Supplemental Fig. SE,E’: *hs*-flp/+; *act5C*-FRT-yellow-FRT-Gal4/+; UAS-SynthAntp long linker-eGFP/+
Supplemental Fig. S7F,F’: *hs*-flp/+; *act5C*-FRT-yellow-FRT-Gal4, UAS-eGFP/+
Supplemental Fig. S7G,G’: *hs*-flp/+; *act5C*-FRT-yellow-FRT-Gal4, UAS-eGFP/+; *Antp* P1-*lacZ*/+
Supplemental Fig. S7H,H’: *hs*-flp/+; UAS-Antp^{RNAi}/+; Antp-eGFP (MiMIC)/*act5C*-FRT-CD2-FRT-Gal4, UAS-mRFP1(NLS)
Supplemental Fig. S9A,A’: *hs*-flp/+; *act5C*-FRT-yellow-FRT-Gal4/+; UAS-SynthAntp short linker-eGFP/+
Supplemental Fig. S9B,B’,G,G’: *hs*-flp/+; *act5C*-FRT-yellow-FRT-Gal4/+; UAS-SynthAntp short linker-eGFP/*Antp* P1-*lacZ*
Supplemental Fig. S9C,C’,H,H’: *hs*-flp/+; *act5C*-FRT-yellow-FRT-Gal4/+; UAS-Antp short linker (full-length, untagged)/*Antp* P1-*lacZ*
Supplemental Fig. S9D,D’: *hs*-flp/+; *Dll*-Gal4 (MD23)/+; UAS-Antp short linker (full-length, untagged), UAS-mRFP1(NLS)/*Antp* P1-*lacZ*
Supplemental Fig. S9E-F’: *hs*-flp/+; *ubi*-FRT-mChery-FRT-Gal4/+; Antp-eGFP (MiMIC)/UAS-Antp short linker (full-length, untagged)
Supplemental Fig. S9I,I’: *ptc*-Gal4/+; UAS-SynthAntp long linker-eGFP/*Antp* P1-*lacZ*
Supplemental Fig. S9J,J’: *ptc*-Gal4/+; UAS-SynthAntp short linker-eGFP/*Antp* P1-*lacZ*
Supplemental Fig. S12A-B’: *ubi*-mRFP1(NLS)/+ or y; Antp-eGFP (MiMIC)/+
Supplemental Fig. S13B,C: *Dll*-Gal4 (MD23)/+; UAS-mCitrine-SynthScr/+
Supplemental Fig. S14A,D,D’: *MS243*-Gal4/+; UAS-SynthAntp long linker-eGFP/*Dr* or *MS243*-Gal4/+; UAS-SynthAntp short linker-eGFP/*Dr*
Supplemental Fig. S14B,F,F’: *MS243*-Gal4/+; UAS-mCitrine-SynthScr/+
Supplemental Fig. S14C,I,I’: *Dll*-Gal4 (MD23)/+; UAS-mCitrine-SynthScr/+
Supplemental Fig. S14E,E’: *ptc*-Gal4/+; UAS-SynthAntp long linker-eGFP/+
Supplemental Fig. S14F,F’: *MS243*-Gal4/+; UAS- mCitrine-SynthScr/+
Supplemental Fig. S14G,G’: *ptc*-Gal4/+; UAS- mCitrine-SynthScr/*Antp* P1-*lacZ*
Supplemental Fig. S14H,H’: *Dll*-Gal4 (MD23)/+; UAS-mCitrine-SynthScr/*Antp* P1-*lacZ*
Supplemental Fig. S14J,J’: *MS243*-Gal4/+; UAS-eGFP/+

Table S1. Phenotypic classes of antenna-to-tarsus transformations for each Gal4-driver used

Phenotypic classes	Number of adults quantified	Ectopic bristles in A3 segment	Mild transformation in A3/Arista	Moderate transformation in A3/Arista	Strong transformation in A3/Arista	No abnormal antenna
69B-Gal4	n=81	76.5%	21%	0%	0%	3.5%
Dll-Gal4 (MD713)	n=93	64.5%	30%	3%	0%	2.5%
ptc-Gal4	n=79	50.5%	45%	4.5%	0%	0%
Dll-Gal4 (MD23)	n=107	0%	0%	13%	87%	0%

Table S2. Parameters used for simulating the stochastic model of *Antp* expression

Parameter	μ_X	η_X^2	μ_O	η_O^2	a	b	τ	k	γ_A	γ_R	λ_A	ρ_A	ρ_R	α	β	γ_S	μ_S	τ_S
Unit	–	–	–	–	–	–	h	min^{-1}	min^{-1}	min^{-1}	min^{-1}	–	–	–	–	–	–	h
Fig. 5 H, I, J	$1.5e3$	0.3	–	–	18	12	12^a	1	$1.0e-8$	$1.6e-7$	150	0.8	1.5	2	2	–	–	–
Fig. 5 K, L	$1.5e3$	0.3	–	–	18	12	12	1	0.25	1	150	0.8	1.5	2	$1-1000$	–	–	–
Fig. 6 D, E[b]	$1.5e3$	0.3	$6e4$	0.3	18	12	12	1	0.25	0	150	0.8	1.5	2	2	–	–	–
Fig. 6 G, H[c]	$1.5e3$	0.3	$4e4$	0.3	18	12	12	1	0.25	0	150	0.8	1.5	2	2	–	–	–
Fig. 6 L, M[d]	$6e2$	0.35	–	–	18	12	12	1	0.25	1	150	0.8	1.5	2	2	$1e-4$	$6e4$	2

[a]Experimentally determined value from (Dworkin et al., 2007)
[b]Overexpression of X_R
[c]Overexpression of X_A
[d]Overexpression of external repressor

Development • Supplementary information

Supplemental references

Affolter, M., Percival-Smith, A., Muller, M., Leupin, W. and Gehring, W. J. (1990). DNA binding properties of the purified Antennapedia homeodomain. *Proc Natl Acad Sci U S A* **87**, 4093-4097.

Bhatia, S., Bengani, H., Fish, M., Brown, A., Divizia, M. T., de Marco, R., Damante, G., Grainger, R., van Heyningen, V. and Kleinjan, D. A. (2013). Disruption of autoregulatory feedback by a mutation in a remote, ultraconserved PAX6 enhancer causes aniridia. *Am J Hum Genet* **93**, 1126-1134.

Duncan, D., Kiefel, P. and Duncan, I. (2010). Control of the spineless antennal enhancer: direct repression of antennal target genes by Antennapedia. *Dev Biol* **347**, 82-91.

Dworkin, I., Lee, W., McCloskey, F. and Larsen, E. (2007). Complex genetic interactions govern the temporal effects of Antennapedia on antenna-to-leg transformations in Drosophila melanogaster. *J Genet* **86**, 111-123.

Engstrom, Y., Schneuwly, S. and Gehring, W. J. (1992). Spatial and Temporal Expression of an Antennapedia Lac Z Gene Construct Integrated into the Endogenous Antennapedia Gene of Drosophila-Melanogaster. *Dev Genes Evol* **201**, 65-80.

Gillespie, D. T. (2007). Stochastic simulation of chemical kinetics. *Annu Rev Phys Chem* **58**, 35-55.

Halford, S. E. and Marko, J. F. (2004). How do site-specific DNA-binding proteins find their targets? *Nucleic Acids Res* **32**, 3040-3052.

Keegan, L. P., Haerry, T. E., Crotty, D. A., Packer, A. I., Wolgemuth, D. J. and Gehring, W. J. (1997). A sequence conserved in vertebrate Hox gene introns functions as an enhancer regulated by posterior homeotic genes in Drosophila imaginal discs. *Mechanisms of development* **63**, 145-157.

Muller, C. B., Loman, A., Pacheco, V., Koberling, F., Willbold, D., Richtering, W. and Enderlein, J. (2008). Precise measurement of diffusion by multi-color dual-focus fluorescence correlation spectroscopy. *Epl-Europhys Lett* **83,** 46001.

Papadopoulos, D. K., Resendez-Perez, D., Cardenas-Chavez, D. L., Villanueva-Segura, K., Canales-del-Castillo, R., Felix, D. A., Funfschilling, R. and Gehring, W. J. (2011). Functional synthetic Antennapedia genes and the dual roles of YPWM motif and linker size in transcriptional activation and repression. *Proc Natl Acad Sci U S A* **108**, 11959-11964.

Rao, C. V. and Arkin, A. P. (2003). Stochastic chemical kinetics and the quasi-steady-state assumption: Application to the Gillespie algorithm. *J Chem Phys* **118**, 4999-5010.

Ryoo, H. D. and Mann, R. S. (1999). The control of trunk Hox specificity and activity by Extradenticle. *Genes Dev* **13**, 1704-1716.

Sela, I. and Lukatsky, D. B. (2011). DNA sequence correlations shape nonspecific transcription factor-DNA binding affinity. *Biophys J* **101**, 160-166.

Soltani, M., Bokes, P., Fox, Z. and Singh, A. (2015). Nonspecific transcription factor binding can reduce noise in the expression of downstream proteins. *Phys Biol* **12**, 055002.

Vukojevic, V., Heidkamp, M., Ming, Y., Johansson, B., Terenius, L. and Rigler, R. (2008). Quantitative single-molecule imaging by confocal laser scanning microscopy. *Proc Natl Acad Sci U S A* **105**, 18176-18181.

Vukojevic, V., Papadopoulos, D. K., Terenius, L., Gehring, W. J. and Rigler, R. (2010). Quantitative study of synthetic Hox transcription factor-DNA interactions in live cells. *Proc Natl Acad Sci U S A* **107**, 4093-4098.

Zechner, C., Ruess, J., Krenn, P., Pelet, S., Peter, M., Lygeros, J. and Koeppl, H. (2012). Moment-based inference predicts bimodality in transient gene expression. *Proc Natl Acad Sci U S A* **109**, 8340-8345.

Development • Supplementary information
